# Prompt and Long-Lived Anti-Kasha Emission from Organic Dyes

**DOI:** 10.3390/molecules26226999

**Published:** 2021-11-19

**Authors:** Daniele Malpicci, Elena Lucenti, Clelia Giannini, Alessandra Forni, Chiara Botta, Elena Cariati

**Affiliations:** 1 Dipartimento di Chimica, Università degli Studi di Milano and INSTM RU, Via Golgi 19, 20133 Milano, Italy; daniele.malpicci@unimi.it (D.M.); clelia.giannini@unimi.it (C.G.); 2 Institute of Chemical Sciences and Technologies “Giulio Natta” (SCITEC) of CNR and INSTM RU, Via Golgi 19, 20133 Milano, Italy; elena.lucenti@scitec.cnr.it; 3 Institute of Chemical Sciences and Technologies “Giulio Natta” (SCITEC) of CNR and INSTM RU, Via Corti 12, 20133 Milano, Italy; chiara.botta@scitec.cnr.it

**Keywords:** anti-Kasha fluorescence, anti-Kasha phosphorescence, organic dyes

## Abstract

Anti-Kasha behavior has been the subject of intense debate in the last few years, as demonstrated by the high number of papers appearing in the literature on this topic, dealing with both mechanistic and applicative aspects of this phenomenon. Examples of anomalous emitters reported in the last 10 years are collected in the present review, which is focused on strictly anti-Kasha organic molecules displaying radiative deactivation from *S_n_* and/or *T_n_*, with *n* greater than 1.

## 1. Introduction

In its original formulation, the Kasha rule states that for complex organic molecules, “regardless of which electronic state of a given multiplicity is excited […], the emitting electronic level of a given multiplicity is the lowest excited level of that multiplicity” [1]. This rule originates from the widespread feature that the E_1_-E_0_ energy gap is much larger than the E_n_-E_n−1_ (*n* > 1) ones and the energy gap law. According to this law, the smaller the energy gap between two electronic states is, the more probable is the nonradiative deactivation (by IC and ISC) between them. In the condensed phase, exceptions to Kasha’s rule are possible under various circumstances. In particular, two prototype mechanisms are responsible for fluorescence from S_2_: (i) when the energy gap between S_1_ and S_2_ is large and the oscillator strength (*f*) of the S_0_-S_2_ transition is large, fluorescence from S_2_ becomes competitive, a mechanism observed for example for azulene and its derivatives [2,3,4,5] and thioketones [6,7,8], the difference between the two families being the (π, π*) character of S_1_ for the former and (*n*, π*) for the latter; (ii) when the S_2_-S_1_ energy gap and *f* of the S_0_-S_1_ transition are very small but *f* of the S_0_-S_2_ transition is large, fluorescence from S_2_ is observed due to thermal population from S_1_, a mechanism observed for example for ovalene [9]. Moreover, fluorescence from higher S_n_ (*n* > 2) can be observed when energy separation between the levels is favorable (necessary condition) and the S_n_-S_1_ internal conversion is prohibited based on symmetry grounds (sufficient condition) [10]. Anti-Kasha phosphorescence from high-energy triplet levels is of course less observed. It may be favored by either an easy ISC (by both El-Sayed and energy gap considerations) to T_2_ level of proper symmetry, different from the T_1_ one so as to give low T_2_-T_1_ IC efficiency, or T_2_ thermal population from T_1_.

Currently, it is proven that the Kasha rule applies to most of the emissive organic molecules, and despite the increased instrumental sensitivity with respect to that available when Kasha made his observation, it is difficult to recognize exceptions. However, “anti-Kasha” emissions represent interesting cases for both fundamental knowledge and application aspects. In fact, anti-Kasha emitters do not dissipate through IC the excess of electronic energy, which can therefore be exploited in practical applications, such as improved luminescent quantum efficiency, tunable emission colors and lifetimes. The interest in this subject is demonstrated by the increasing number of works appearing in the literature in the last few years. In this regard, in 2012, Itoh reviewed works produced until 2011 on traditional anti-Kasha emitters such as azulene and its derivatives, aromatic acenes, polyenes, thioketones, metalloporphyrins, aromatic carbonyl compounds, quinones and halogenated aromatic compounds [9]. The reported examples comprise emission from higher singlet and triplet levels.

Anomalous emission includes a large number of photophysical phenomena, among which anti-Kasha behavior represents a very limited subset that manifests either through single or multiple emissions [11]. The first case may be particularly difficult to discern because low-lying dark excited levels are silent to many experimental investigations. Importantly, when more than one band is displayed by a single molecule due to structural rearrangement and/or chemical transformation, such as “excited state conjugation enhancement” (ESCE), “twisted intramolecular charge transfer” (TICT), “excited state intramolecular proton transfer” (ESIPT) and “optically triggered counterion migration” (OTCM) [11], dual or multiple emissive behavior is to be used instead of anti-Kasha behavior. Actually, a few examples of ESCE, TICT, ESIPT and OTCM associated with anti-Kasha behavior exist [12] since the reorganization occurs starting from a higher excited state in the molecule. However, such examples will not be covered in the present review. Even those cases where various emissions are due to the molecule and its aggregates in the same phase (solution, amorphous or crystalline solids) have to be described as Kasha emitters. Of course, trivial mistakes such as the presence of traces of impurities which can be responsible for the appearance of multiple emission bands should be carefully checked before claiming an anti-Kasha behavior [13,14].

Here we collect works reporting “rigorous” anti-Kasha emitters that have appeared in the literature since 2011, starting from the seminal analysis from Itoh [9]. By “rigorous”, we mean systems that radiatively deactivate from S_n_ or T_n_ (*n* > 1). In fact, in the literature, some works report as anti-Kasha a behavior that instead could be better described as anti-Vavilov [14,15]. According to the Vavilov rule, the intensity of the dye emissions is independent of excitation wavelength. 

Another important caveat should be presented. Since anti-Kasha behavior is hardly recognizable through an exclusively experimental approach, in particular when states below the emissive S_n_ or T_n_ (*n* > 1) ones are silent, quantum-chemical computational studies acquire a key role in establishing the presence and the origin of anomalous emission. Owing to the high computational costs associated with accurate *ab initio* techniques (e.g., CASPT2 and MRCI), most theoretical investigations aimed at predicting electronic spectra of complex molecules are performed within the TDDFT approach. The performance of this method was always deemed highly reliable for predicting non-charge-transfer excitations, while it is well known that exchange-correlation functionals generally used for ground-state calculations, such as those based on the generalized gradient approximation (GGA) or the hybrid GGA functionals (typically the popular B3LYP one) do not allow for sufficient accuracy to study CT excited states [16,17,18,19,20,21]. Very few exceptions (for example, the PBE0 functional) have been recognized [22]. Recently, the development of the so-called range-separated hybrid functionals (including CAM-B3LYP, ωB97X and ωB97XD), where the exchange term is split into long- and short-range [23,24,25,26,27] and the range-separation parameter can be suitably determined by a self-consistent procedure [28], has allowed for easy resolution of the CT problem for many systems of practical interest [29,30]. Additional functionals have been developed since, such as the hybrid meta-GGA ones (including Mo6-2X) whose performance in electronic spectra calculations has been assessed [31]. It is therefore to be noted that the cases of anti-Kasha behavior involving CT excited states collected in the present review, especially those where TDDFT calculations have been performed using inadequate functionals, could be confuted by more reliable approaches.

This manuscript is organized by different families of molecules, including azulenes, cyclic triimidazoles, 1,2-diphenylphenanthroimidazole derivatives, cyanines, thiophenes and carbazoles.

## 2. Azulenes

**Scheme 1:**
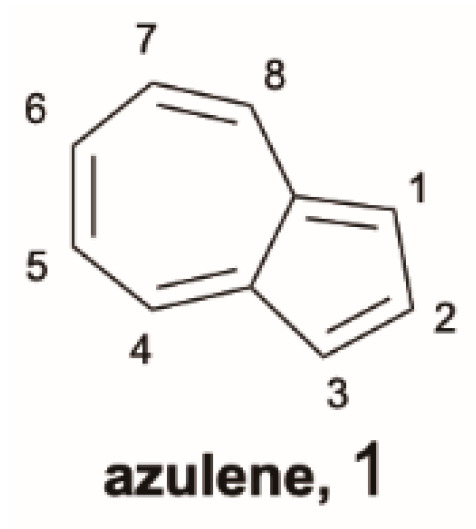
Chemical structure of compound **1**.

Azulene, **1** (Scheme 1), can be regarded as the forefather of organic molecules displaying anti-Kasha behavior, namely a fluorescent emission from the S_2_ excited state [9,32].

**Figure 1:**
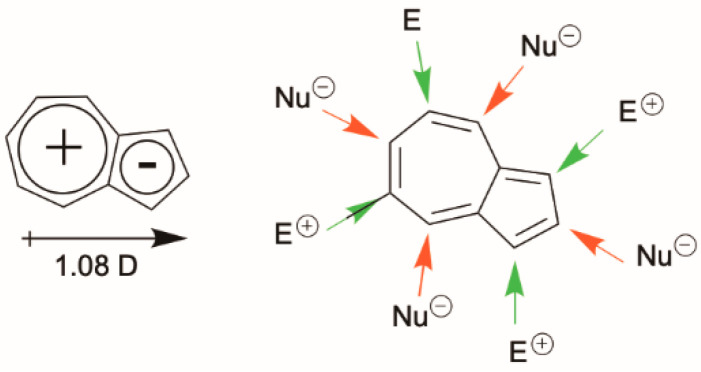
Polarized resonance structure of azulene with the dipole moment (**left**) and reactivity of each position of azulene (**right**). Adapted with permission from [33], Copyright 2021, American Chemical Society.

This feature derives from the unique molecular structure of azulene, different from other traditional aromatic compounds and characterized by an electron-rich five-membered ring fused with an electron-poor seven-membered ring with HOMO density localized mainly on the odd positions and LUMO density located on the even positions [33] (Figure 1). This leads to an unusually low-lying first electronic excited state (S_1_), favoring a large S_2_-S_1_ energy gap (>1.2 eV) and, according to the energy-gap law, resulting in fluorescence from S_2_ rather than IC to S_1_. Similar emissive behavior is found for variously substituted azulenes, polyazulenes and pseudoazulenes (having a −CH=CH− group in the heptagonal ring of azulene replaced by a heteroatom). It has been shown that HOMO and LUMO energy levels can be modified by proper choice of electron-donor or electron-withdrawing functional groups. In particular, the insertion of electron-donor substituents at the 1, 3, 5 or 7 ring positions usually increases the S_2_-S_1_ gap while electron-withdrawing substituents have the opposite effect. A reverse behavior is found for substitutions at the 2, 4, 6 and 8 positions, resulting in an S_2_-S_1_ energy gap increased by electron-withdrawing groups and lowered by electron-donor substituents. In some cases, for derivatives characterized by a relatively small S_2_-S_1_ energy gap, IC to S_1_ becomes competitive with fluorescence from S_2_, and fluorescence from both excited states is observed [9,32]. Azulene and a series of its derivatives have been the subject of accurate theoretical benchmark studies to validate state-of-the-art computational protocols predicting anti-Kasha photoluminescence [34,35].

Due to these intriguing photophysical properties, there is a growing interest in the development of azulene-based materials for optoelectronic applications, even though the preparation of new derivatives is hampered by multistep and low-yield synthetic procedures [32,33,36].

**Scheme 2:**
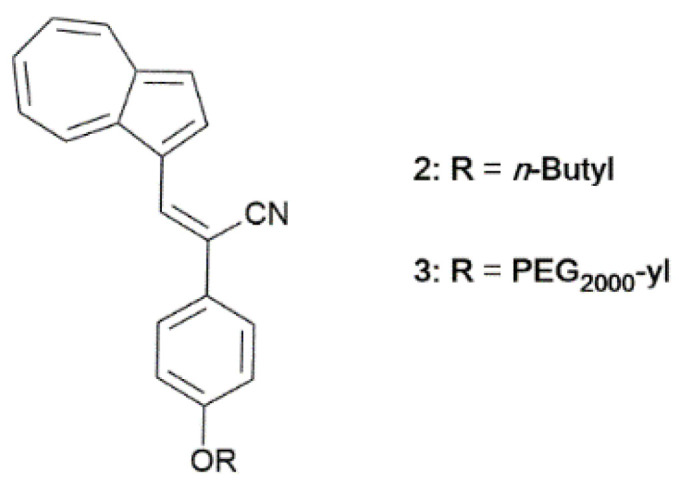
Chemical structure of compounds **2** and **3**.

**Figure 2:**
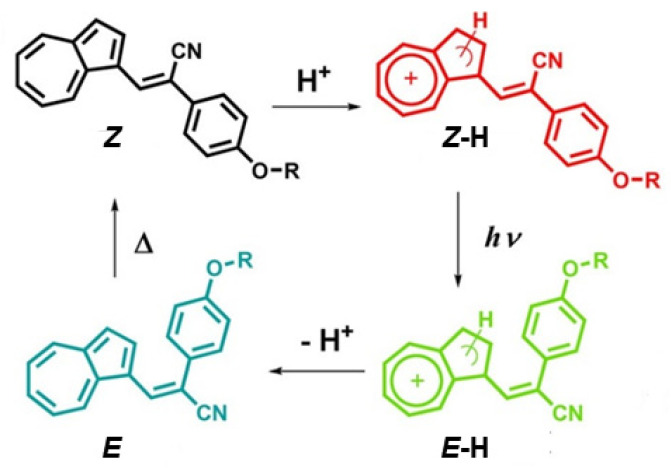
(**Top**) Chemical structures of the cyanostyryl-modified azulenyl compounds **2** and **3**. (**Bottom**) Protonation-assisted photoisomerization process. Adapted with permission from [37].

**Figure 3:**
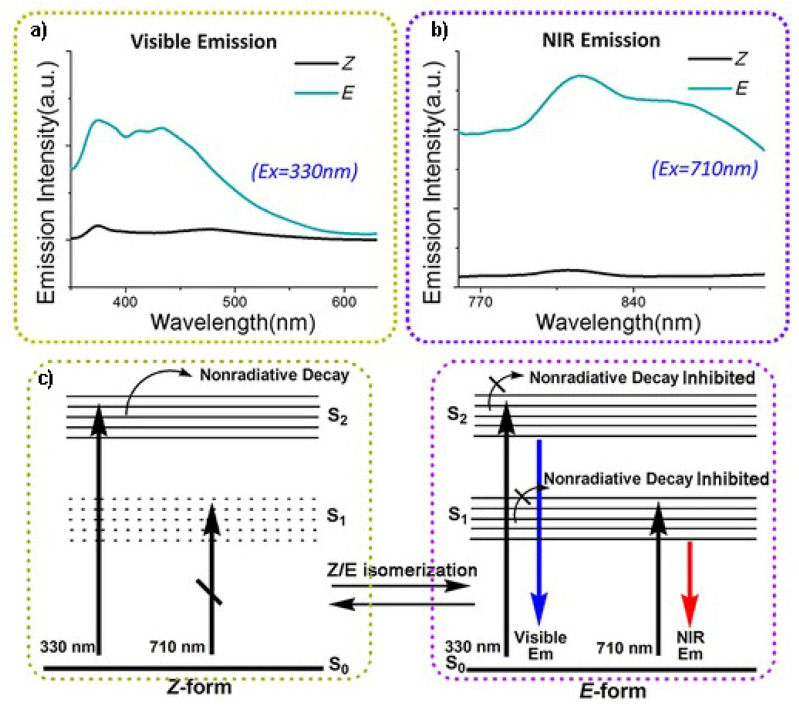
Emission spectra of **3** in Z- and E-form in water (5 × 10^−5^ mol L^−1^) at RT. (**a**) λ_ex_ = 330 nm, scanning range = 350–900 nm; (**b**) λ_ex_ = 710 nm, scanning range = 730–900 nm. (**c**) Proposed mechanism for the tuning of the dual pathway luminescence in Z- and E-form. Adapted with permission from [37].

In this regard, Zhou et al. [37] reported on two azulene derivatives, **2** and **3** (Scheme 2), substituted at the 3 position with cyanostyryl moieties that, under UV or visible irradiation in acidic conditions, photoisomerize from Z- to E-form (Figure 2). The process is accompanied by a strong modification of the photoluminescent features. In particular, nonradiative decays are inhibited by sterical restrictions going from the not emissive Z-isomer to the E-isomer. Moreover, the E-form displays dual luminescence with a major emission band at around 480 nm (assigned to an S_2_-S_0_ transition) and an NIR emission at around 800 nm (attributed to a radiative decay from S_1_, Figure 3). The conversion between the two isomers is reversible, and the E-isomer can be converted into the Z-isomer by simply heating the sample at a temperature above 65 °C. 

**Scheme 3:**
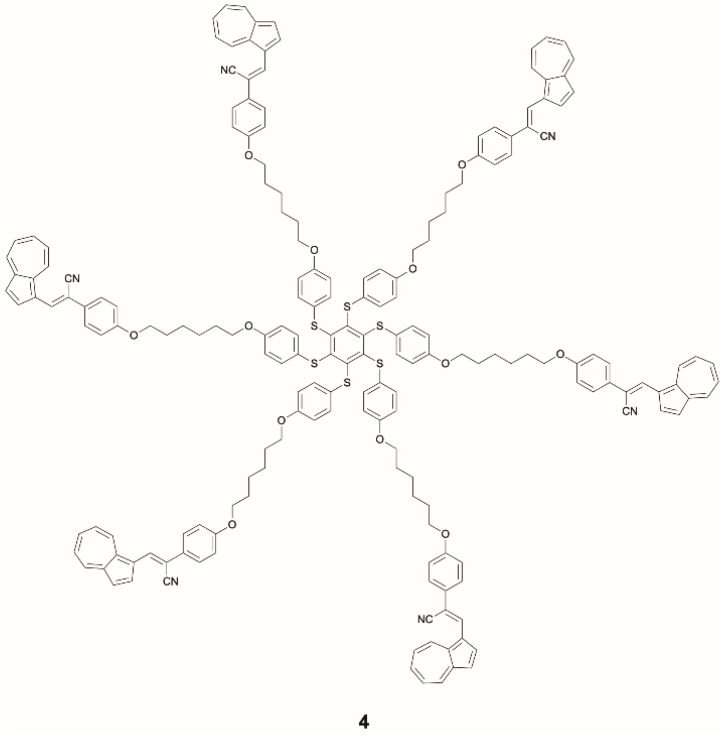
Chemical structure of compound **4**.

The same research group investigated the photophysics of compound **4** (Scheme 3) in which dual Vis and NIR emissive cyanostyryl-modified azulene moieties are inserted onto a hexathiobenzene core [38]. With respect to its cyanostyryl–azulene precursor, **4** displays an increased anti-Kasha emission at 480 nm due to a FRET process from the hexathiobenzene to the azulene. The emission intensity of **4** can be further enhanced in tetrahydrofuran (THF)/H_2_O solvent mixtures by an AIE effect, thus leading to a total 15-fold amplification of the QY of this material compared to its precursors. In addition, **4** is a rare example of multiwavelength anti-Kasha emissions since it displays, in addition to the visible S_2_-S_0_ fluorescence at 480 nm, a UV emission at 360 nm, generated by radiative decay from the higher S_3_ excited state. Interestingly, these emissive features are retained in PMMA films, opening the way to possible applications of solid-state anti-Kasha materials. 

**Scheme 4:**
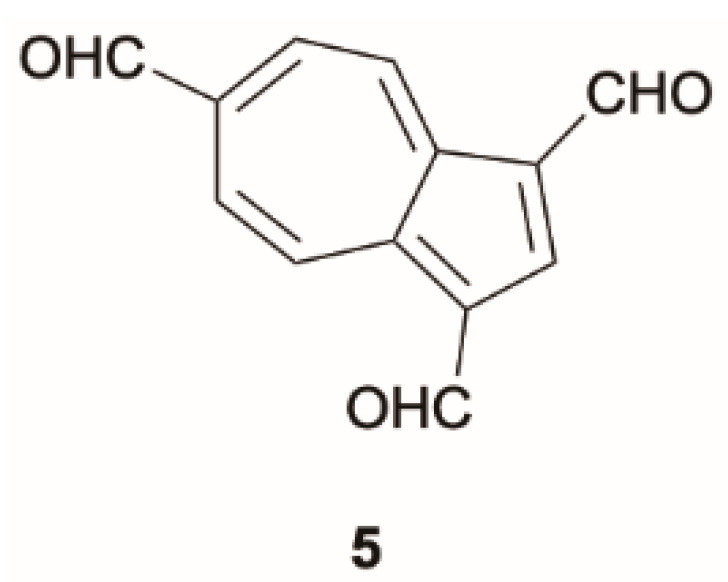
Chemical structure of compound **5**.

**Figure 4:**
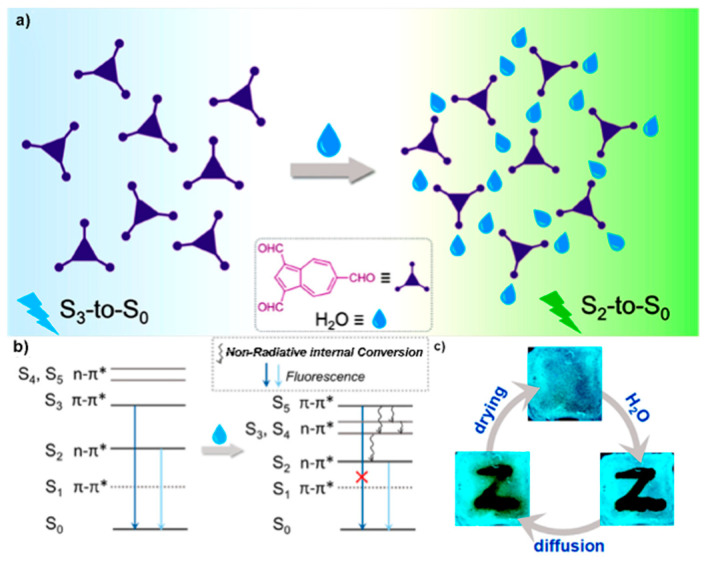
(**a**) Proposed process for the formation of intermolecular H-bonding of compound **5** upon addition of a small amount of H_2_O. (**b**) Proposed mechanism accompanied by an anti-Kasha luminescent conversion for the tuning of the **5** luminescence in DMF/H_2_O mixtures. (**c**) The writing of “Z” character with H_2_O onto the **5**-doped PVA/PAAS film on a quartz substrate and the process of the three-state recyclable (photographs were taken under 365 nm UV light). Adapted with permission from [39], Copyright 2018, American Chemical Society.

In an extension of their research, Zhou et al. [39] reported on a series of mono-, di- and triformyl-substituted azulenes, among which the triformyl derivative **5** (Scheme 4) shows a remarkable dual fluorescence (photoluminescence QY, Φ, up to 10%) in DMF solution. On the basis of experimental and theoretical investigations, the latter based on TDDFT calculations at the B3LYP/6-31G(d) level of theory, the photoluminescent behavior of **5** was attributed to concomitant radiative deactivations from the upper S_3_ and S_2_ energy levels with (π, π*) and (*n*, π*) character, respectively, and having an energy gap of 0.35 eV which guarantees a slow IC. In the presence of water, intermolecular H-bonds between the aldehydic groups and water result in the red shift of the emission and in the quenching of the S_3_ deactivation path due to the stabilization of S_4_ and S_5_ (*n*, π*) levels (see Figure 4b). Tunable emission color, from blue to cyan and green, by H-bonding control was demonstrated both in solution and in the solid state by using hydrophilic polymers such as polyvinyl alcohol (PVA) and polyacrylic acid sodium salt (PAAS) as matrices to ensure a sufficient moisture sensitivity and the realization of a visual sensor for moisture (Figure 4c).

**Scheme 5:**
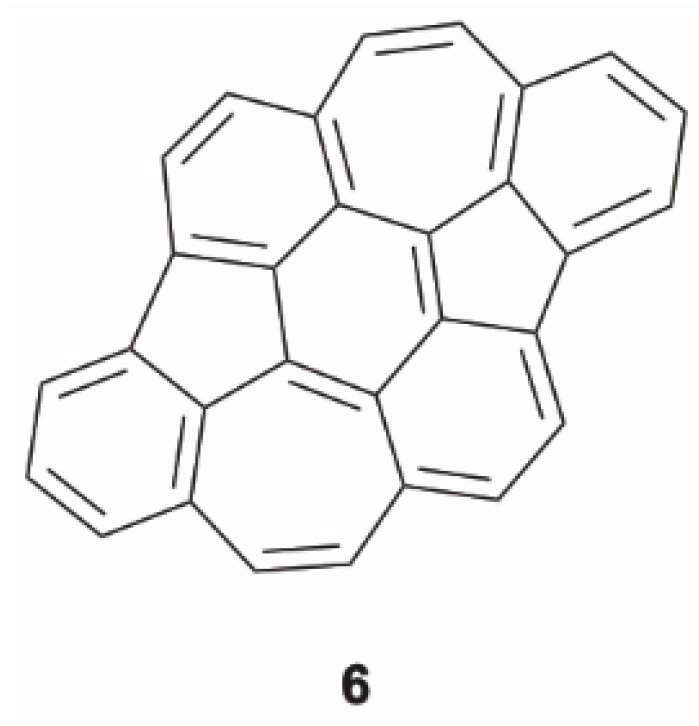
Chemical structure of compound **6**.

Zhang et al. [40] reported on the synthesis of dicyclohepta[*ij*-*kl*,*uvwx*]rubicene, **6** (Scheme 5), a polycyclic aromatic hydrocarbon containing two coplanar pentagons and heptagons constituting two formal azulene moieties. The presence of these conjugated rings ensures thermal and air stability together with planarity, as confirmed by crystal structure analysis. A combined photophysical and theoretical investigation revealed the presence in THF solution of two emission bands at 670 and 400 nm which were assigned, according to CASPT2/6-31G(d)/(12,12) calculations based on CASSCF orbitals, to S_1_-S_0_ and S_2_-S_0_ radiative decays, respectively. The anti-Kasha emission was established to originate mainly from a contribution of the two formal azulene units present in the molecular structure. 

**Scheme 6:**
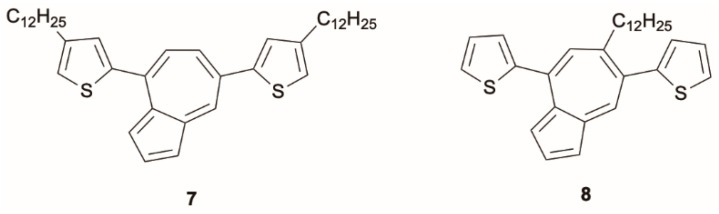
Chemical structure of compounds **7** and **8**.

**Figure 5:**
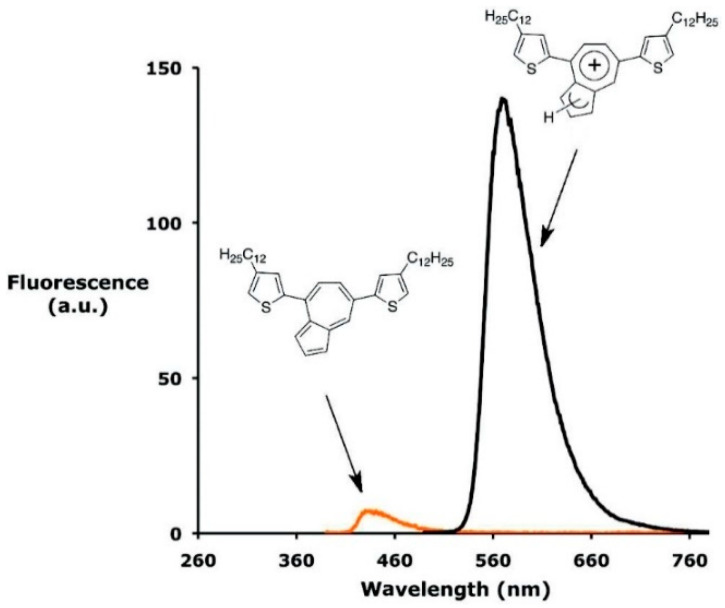
Fluorescence measurements in dichloromethane for neutral oligomer **7** and after protonation to give **7H^+^**. Reprinted with permission from [41], Copyright 2011, American Chemical Society.

Based on the observation that protonation of azulene can result in a tropylium form with intensified fluorescence due to the suppression of anti-Kasha emission in favor of the S_1_ one, Amir et al. [41] studied 5,5′-(azulene-4,7-diyl)bis(3-dodecylthiophene), **7**, and 2,2′-(6-dodecylazulene-4,7-diyl)dithiophene, **8** (Scheme 6). The two compounds can, in fact, undergo a reversible protonation/deprotonation process by treatment with trifluoroacetic acid (TFA) and triethylamine (NEt_3_). The acidochromic switch can be easily followed by fluorescent spectroscopy (see Figure 5) revealing a red shift and a remarkable increase in emission intensity upon protonation and the quenching of the signal by deprotonation with the base. 

**Scheme 7:**
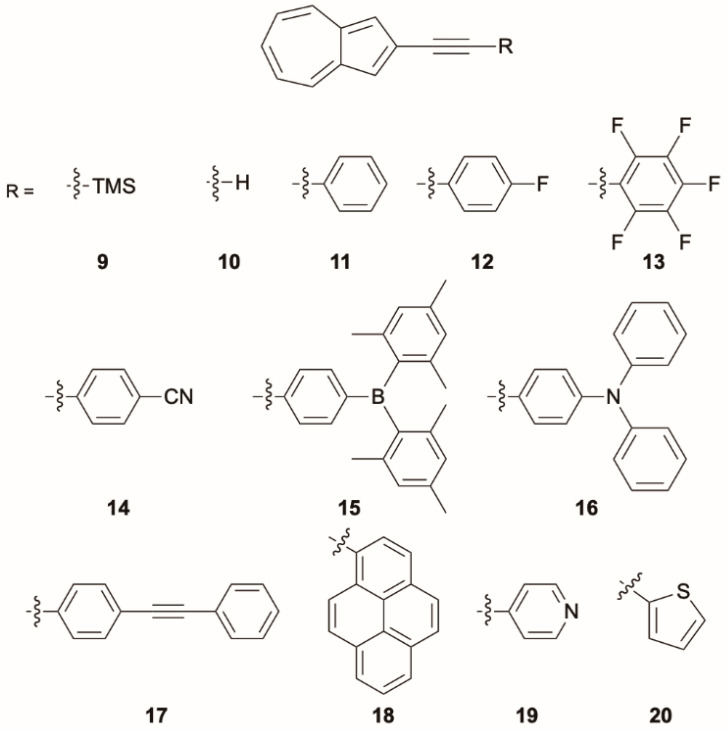
Chemical structure of compounds **9**–**20**.

A similar behavior was reported by Koch et al. [42] for a series of 2-alkynyl azulenes, **9**–**20** (Scheme 7), characterized by a weak fluorescence that can be enhanced and red-shifted by treatment with acids. 

**Scheme 8:**
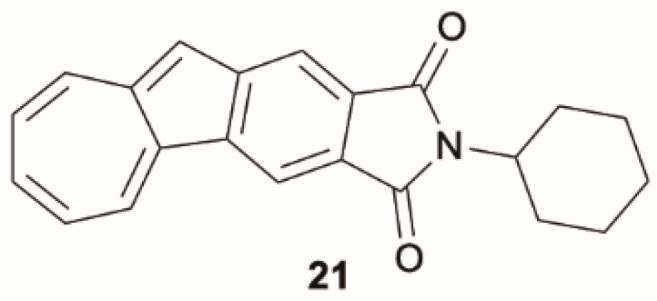
Chemical structure of compound **21**.

Shoij et al. [43] reported on a family of azulene-fused phthalimides, among which derivative **21** (Scheme 8) displays the typical azulene fluorescent anti-Kasha feature. Compound **21** and a series of 2-arylazulenes reported by the same authors [44] show the typical intensification of the S_1_ emission through protonation, leading to suppression of anti-Kasha behavior.

**Scheme 9:**
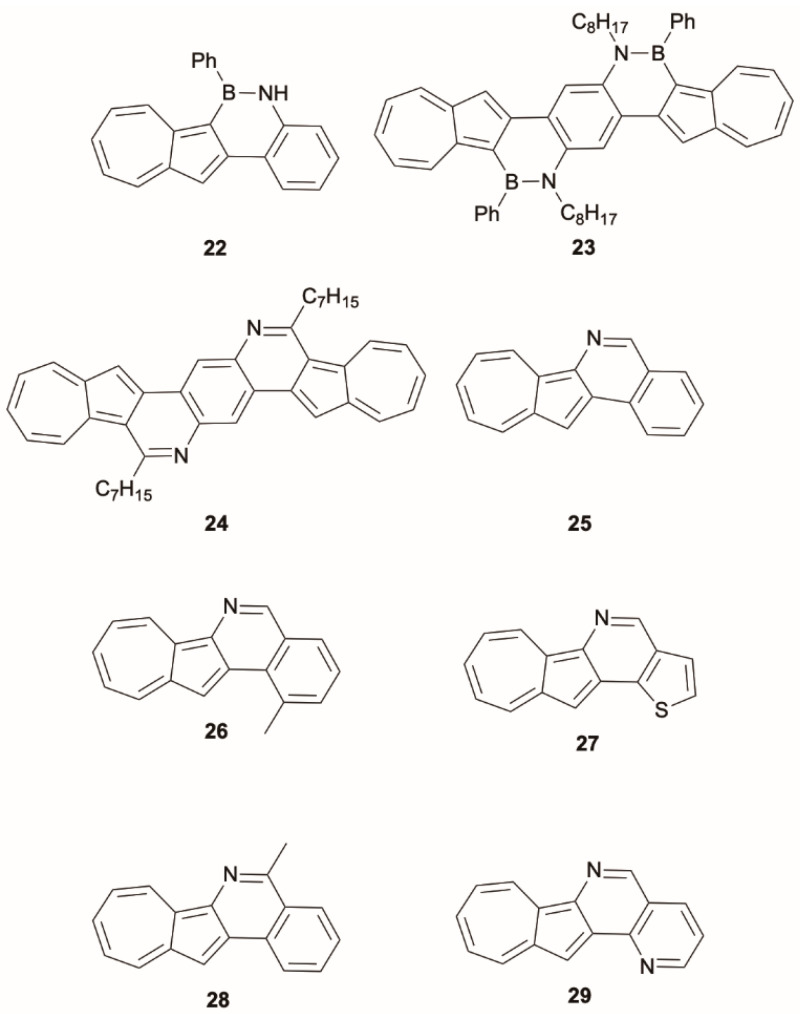
Chemical structure of compounds **22**–**29**.

In the search of new polycyclic heteroaromatics for potential optoelectronic applications, new examples of azulene derivatives showing anti-Kasha behavior were reported by Xin et al. [45], who prepared the first two azulene-based BN-heteroaromatics, **22** (Scheme 9) and **23**, by nitrogen aromatic borylation. Their analysis was supported by TDDFT calculations at the CAM-B3LYP/Def2-TZVP level of theory. The same research group [46] described the reductive cyclization of 1-nitroazulene precursors to produce a series of azulene-pyridine-fused heteroaromatics, **24**–**29**, displaying photophysical features typical of azulene derivatives with a weak emission band originating, based on CAM-B3LYP/6-311G(d,p) TDDFT calculations, from S_2_ or higher excited states and no detectable emission from S_1_. 

## 3. Cyclic Triimidazoles

**Scheme 10:**
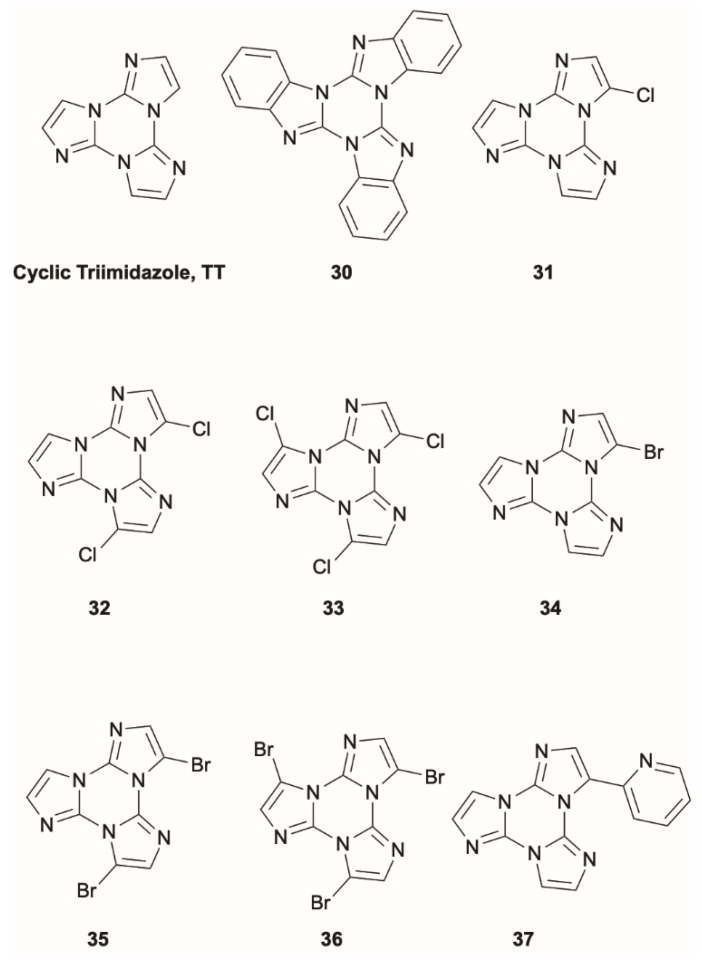
Chemical structure of **TT** and compounds **30**–**37**.

Lucenti et al. [47] investigated an extensive series of “cyclic triimidazole”, **TT** (Scheme 10), derivatives (where **TT** is triimidazo[1,2-*a*:1′,2′-*c*:1″,2″-*e*][1,3,5]triazine) characterized, mainly in the solid state, by an intricated multiemissive behavior of molecular and aggregation-induced origin, including RTP associated with π–π interactions. Within this rich photophysics, for some members of this family, a fluorescent or phosphorescent anti-Kasha behavior was recognized. 

**Figure 6:**
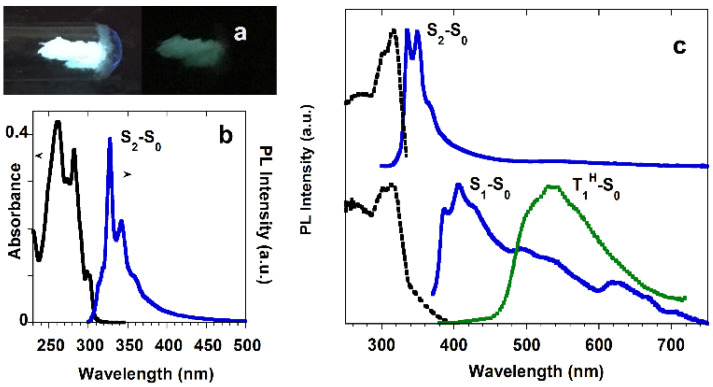
Compound **30**. (**a**) Powders at 77 K with UV irradiation on (**left**) and off (**right**). (**b**) In DCM at RT: absorption and emission (blue line, λ_ex_ = 260 nm). (**c**) Powders at RT: Top: excitation (dashed black line, λ_em_ = 348 nm) and emission (blue line, λ_ex_ = 260 nm). Bottom: excitation (dashed black line, λ_em_ = 408 nm), emission (blue line, λ_ex_ = 370 nm) and phosphorescence (green dotted line, 10 ms delay, window 50 ms, λ_ex_ = 358 nm). Reprinted with permission from [48].

In particular, benzo[4,5]imidazo[1,2-*a*]benzo[4,5]imidazo[1,2-*c*]benzo[4,5]imidazo[1,2-*e*][1,3,5]triazine, **30** (Scheme 10), displays a single fluorescent emission at about 330 nm (Φ = 17%) in diluted DCM solution at RT, which, based on TDDFT calculations at the ωB97X/6-311++G(d,p) level of theory, was associated with deactivation from an S_2_ excited state [48]. This attribution was supported by a calculated substantial S_2_-S_1_ energy gap (0.35 eV) and an S_0_-S_1_ transition with zero oscillator strength owing to the high symmetry (ideally *C*_3h_) of the molecular π-electron system. Intriguingly, solid **30** shows both phosphorescence and dual fluorescence (Φ = 18%) at RT, which can be selectively activated by varying the excitation wavelength (see Figure 6). At 260 nm excitation wavelength, a near-UV fluorescence emission at 350 nm of S_2_-S_0_ origin, similar to that of the chromophore in dilute solution, is observed. By exciting at 370 nm, a structured blue fluorescence at 407 nm superimposed onto the phosphorescent emission at 530 nm is produced. The authors explained the presence of this second fluorescence of S_1_-S_0_ nature, selectively activated only by populating the S_1_ state, by partial loss of the molecular symmetry of the chromophore owing to interchromophoric interactions in the solid state. 

**Figure 7:**
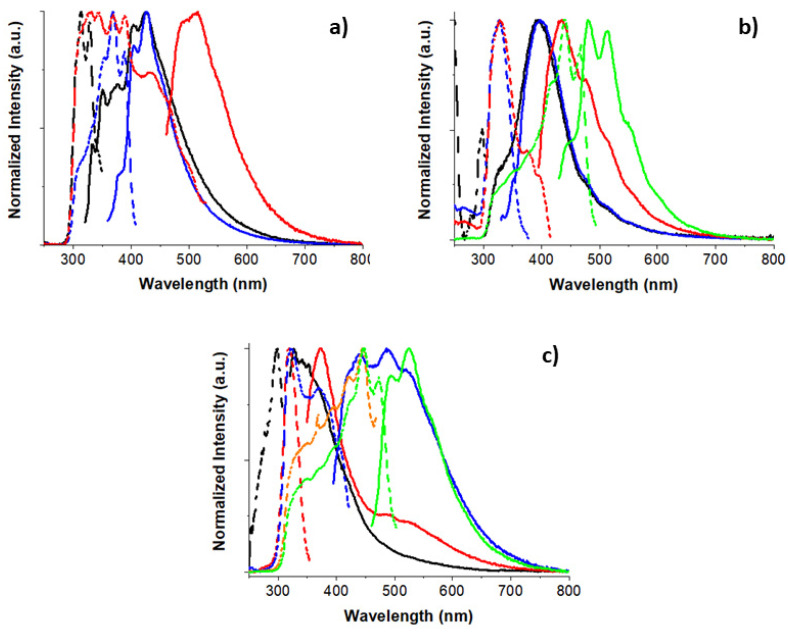
(**a**) Normalized excitation (dotted lines) and emission (solid lines) spectra of **31** crystals at 298 K. Emission: λex = 300 nm (black), λex = 340 nm (blue) and λex = 440 nm (red). Excitation: λem = 371 nm (black), λem = 427 nm (blue) and λem = 550 nm (red). (**b**) Normalized excitation (dotted lines) and emission (solid lines) spectra of **32** crystals at 298 K. Emission: λex = 280 nm (black), λex = 308 nm (blue), λex = 375 nm (red) and λex = 413 nm (green). Excitation: λem = 328 nm (black), λem = 398 nm (blue), λem = 436 nm (red) and λem = 514 nm (green). (**c**) Normalized excitation (dotted lines) and emission (solid lines) spectra of **33** crystals at 298 K. Emission: λex = 300 nm (black), λex = 330 nm (red), λex = 374 nm (blue) and λex = 440 nm (green). Excitation: λem = 327 nm (black), λem = 373 nm (red), λem = 441 nm (blue), λem = 487 nm (orange) and λem = 524 nm (green). Adapted with permission from [49].

Similarly, mono-, di- and trihalogenated **TT** compounds [48,49,50], **31**–**36** (Scheme 10), display dual fluorescence in the solid state due to a large S_n_-S_1_ energy splitting and a low or zero oscillator strength of the S_0_-S_1_ transition. In particular, **31**–**33** (Φ = 24%, 12% and 9%, respectively) are characterized by multiple long-lived emissive components (due to molecular, T_1_ and aggregated forms) in addition to dual fluorescence (one at about 340 nm for the three compounds and the other at about 350, 400 and 370 nm for **31**, **32** and **33**, respectively; see Figure 7), better resolved at low temperature [49]. The two fluorescences, based on analysis of excitation spectra showing a first absorption at 320–330 nm and a second absorption at 300 nm, were assigned to deactivation from S_1_ and S_n_ (*n* > 1), respectively, with an experimental S_2_-S_1_ energy gap in the 0.27–0.38 eV range. This anti-Kasha origin of the dual fluorescence was supported by ωB97X/6-311++G(d,p) DFT/TDDFT calculations which provided an S_1_ state of low oscillator strength (*f* = 0.013, 0.009 and 0 for **31**, **32** and **33**, respectively) due to symmetry reasons and high-energy singlet levels with large oscillator strength (S_3_ and S_4_, *f* > 0.4).

Analysis of the emissive properties of **31**–**33** in solution was precluded by the very low quantum efficiency of the compounds at low concentration. On the other side, DCM solutions of **34**–**36** [48,50] display a weak but recognizable fluorescence with maxima at 328, 380 and 370 nm, respectively (Φ equal to 3% for **34** and almost vanishing for **35** and **36**), which was ascribed, on the basis of ωB97X/6-311++G(d,p) DFT/TDDFT calculations, to a high-energy S_n_ state (*n* = 3; 4, 5; 6, 7 for **34**–**36**, respectively). Again, the absence of the S_1_-S_0_ radiative deactivation channel was explained by the low *f* of the S_0_-S_1_ transition (0.024, 0.016 and 0 for **34**–**36**, respectively) associated with the high symmetry of the **TT** π-electron system, only partially disrupted in **34** and **35**, and to the sizable S_n_-S_1_ energy gap (calculated as 0.43, 0.44 and 0.48 eV for **34**–**36**, respectively).

**Figure 8:**
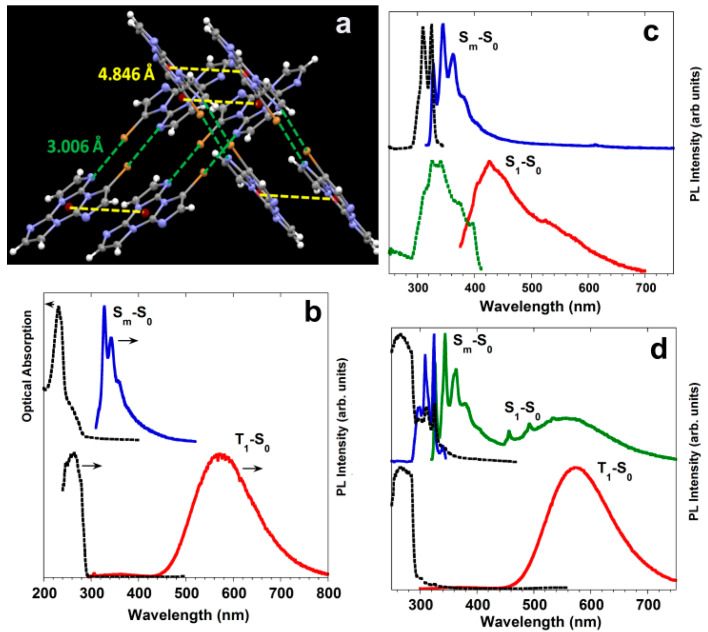
Compound **34**. (**a**) Crystal packing: π-π stacking interactions and Br^…^N XB shown as yellow and green dotted lines, respectively. (**b**) In DCM: top: absorption and emission (λex = 280 nm) at RT; bottom: excitation (black dotted line, λem = 580 nm) and emission (red line, λex = 280 nm) at 77 K. (**c**) Powders at RT: top: excitation (black dotted line, λem = 363 nm) and emission (blue line, λex = 300 nm); bottom: excitation (green dashed line, λem = 429 nm) and emission (red line, λex = 360 nm). (**d**) Powders at 77 K: top: emission (green line, λex = 300 nm) and excitation (blue line, λem = 363 nm; black dashed line, λem = 492 nm); bottom: excitation (black dashed line, λem = 580 nm) and emission (red line, λex = 280 nm). Reprinted with permission from [48].

Interestingly, unlike S_1_ and S_n_ having (π, π*) character, the intermediate S_m_ levels (S_2_, S_2,3_ and S_2–4_ for **34**–**36**, respectively) correspond to forbidden transitions (*f* = 0) with partial (π, σ*) character, where the involved σ orbitals are mainly delocalized on Br atom(s) and C–Br bond(s). Going to the solid state (Φ < 0.1, 14 and <0.1 for **34**–**36**, respectively) (see Figure 8 for **34**), molecular and intermolecular-induced phosphorescences are observed in the spectra of the three compounds. Moreover, while an S_m_-S_0_ emission is visible for **34**–**36** (at 326, 345, 365, 382; 395, 419, 443; 394, 418, 444 nm, respectively), the S_1_-S_0_ one appears only for **34** and **36** (with maxima at 426 and 415 nm, respectively). This latter emission, associated with the distorting packing forces which reduce the symmetry of the molecular π-electron system, is the only emission observed by exciting at high energy (E > S_m_) while dual fluorescence is produced by exciting to S_m_ levels. A possible interpretation was provided by ωB97X/6-311++G(d,p) DFT/TDDFT calculations on the π–π dimer of **36**, which revealed a manifold of S_m_ levels of (π, π*), (π, σ*) and mixed (π, π*)/(π, σ*) character where the latter possess the strongest oscillator strength. It is therefore expected that excitation to S_m_ results in both radiative deactivation (from (π, π*)/(π, σ*) levels) to S_0_ and IC (from (π, π*) levels) to S_1_, while excitation to (π, π*) S_n_ leads to IC to S_1_. In the case of **35**, no mixed S_m_ (π, π*)/(π, σ*) states came out from calculations on its π–π dimer, and the (π, σ*) excitations have *f* < 0.01, suggesting that S_m_ levels have predominant (π, π*) character with *f* greater than that of S_1_. Therefore, irrespective of excitation energy, these (π, π*) S_m_ levels are responsible for the observed single fluorescence.

3-(Pyridin-2-yl)triimidazo[1,2-*a*:1′,2′-*c*:1″,2″-*e*][1,3,5]triazine, **37** (Scheme 10), is the only member of the **TT** family showing anti-Kasha behavior from an excited triplet level [51]. This compound crystallizes in three phases characterized by a highly intricated photophysics comprising multiple fluorescent and phosphorescent bands (Φ = 50%). The dual fluorescence observed at about 350 and 450 nm was associated with two different molecular conformations; therefore, it does not represent an exception to the Kasha rule. On the other side, two (at about 370 and 400 nm) of the four observed phosphorescences were ascribed to molecular origin and interpreted, on the basis of ωB97X/6-311++G(d,p) DFT/TDDFT calculations, as derived from triplet levels of different character. In particular, the high energy contribution resulted from the calculated T_6_ level having (σ/π, π*) character, while the low energy contribution derived from the calculated T_1_ with (π, π*) symmetry. 

## 4. 1,2-Diphenylphenanthroimidazole (PPI) Derivatives

**Scheme 11:**
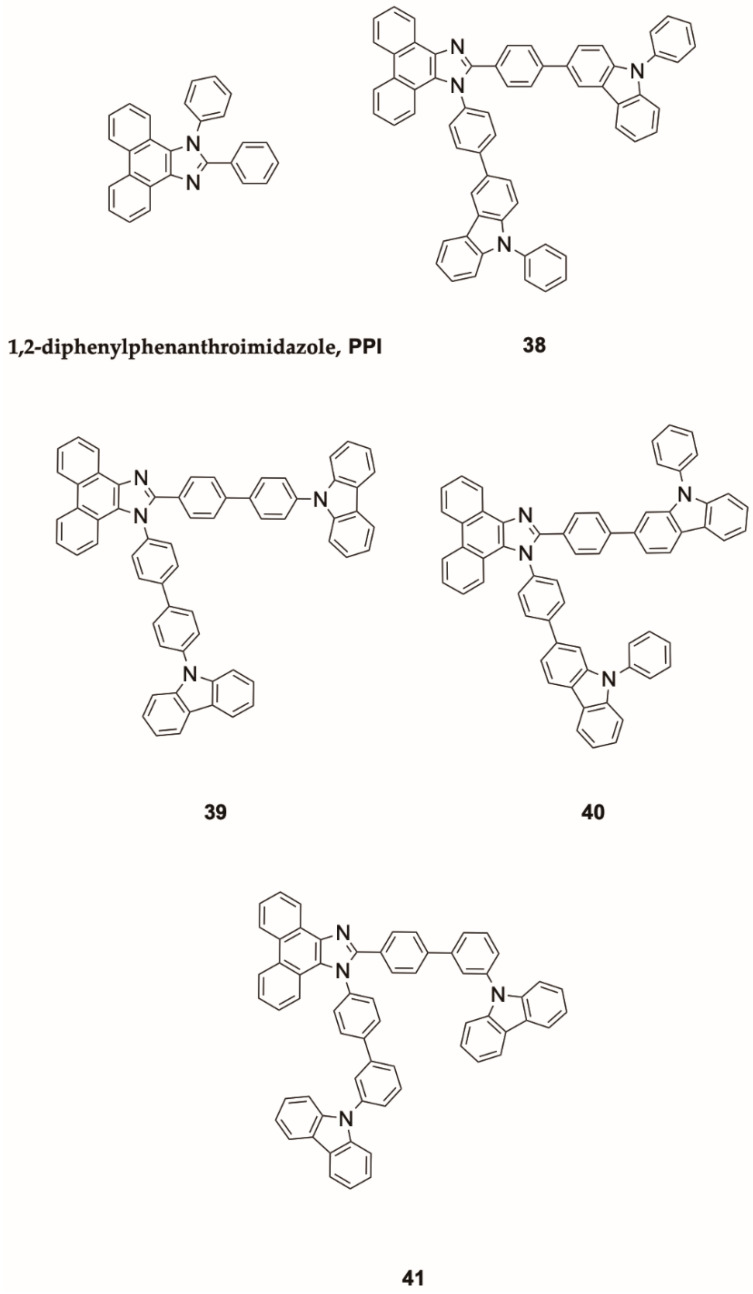
Chemical structure of **PPI** and compounds **38**–**41**.

1,2-Diphenylphenanthroimidazole, **PPI** (Scheme 11), represents an interesting tecton for the preparation of new emissive materials due to its intense blue fluorescence and its bipolar character associated with the presence of two distinct nitrogen atoms in the imidazole ring. Therefore, **PPI** can be used as an electron acceptor when combined with a strong electron-donor group or as an electron donor when combined with an electron-deficient or a weak electron-donor group. By exploiting **PPI** bipolarity, Yang et al. [52] prepared and investigated the symmetrical triads PPI-PCz, **38**–**41** (Scheme 11) (PCz is 9-phenylcarbazole), where **PPI** plays the role of the donor inside the molecule as revealed by femtosecond TA studies. Interestingly, for **38**–**41**, anti-Kasha ICT behavior was reported. 

In diluted DMF solution, **38**–**41** display a fluorescent emission at 420 nm (Φ almost 100%), with a red shift of 55 and 30 nm with respect to that of PCz and **PPI**, respectively. TDDFT calculations on **38**–**41**, performed at the B3LYP/6-311G(d,p) level of theory, revealed for the S_0_-S_2_ transition a higher probability than the S_0_-S_1_ one due to its higher oscillator strength. Moreover, calculations confirmed the S_0_-S_2_ ICT nature with the HOMO mainly located on the **PPI** fragment and the LUMO located on PCz and phenyl moieties. 

**Scheme 12:**
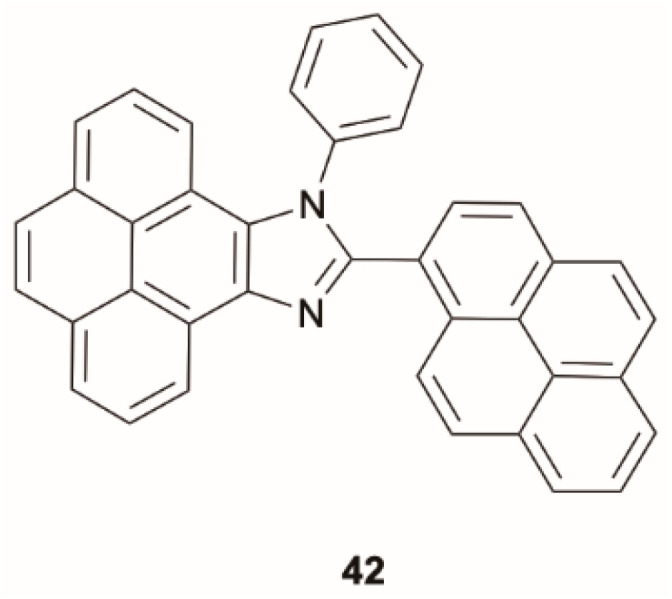
Chemical structure of compound **42**.

Qiao et al. [53] reported on the excitation-dependent and anti-Kasha emissive behavior of 9-phenyl-10-(pyren-1-yl)-9*H*-pyreno[4,5-*d*]imidazole, **42** (Scheme 12).

**Figure 9:**
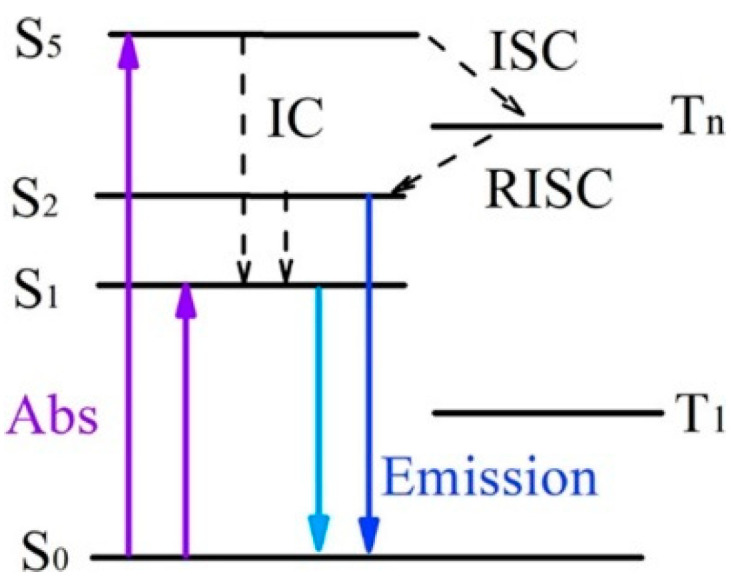
Exciton evolution route. Schematic potential key photophysical processes proposed for anti-Kasha photoemission of **42**. Abs, IC, ISC and RISC represent the absorption, IC, ISC and reverse ISC, respectively. The absorption and emission processes are represented as solid arrow lines. The nonradiative processes are indicated as dashed arrow lines. Reprinted with permission from [53], Copyright 2019, American Chemical Society.

When excited at 350 nm, neat and PMMA blended film of **42** display one fluorescence at 468 nm (τ equal to 4.5 ns). However, at 250 nm excitation wavelength, an additional dominant emission at 392 nm (τ equal to 109.7 μs) is produced together with the 468 nm one. Excitation profiles of the two emissions obtained for the blended film indicated for the low-energy emission the same profile observed in absorption, while the high-energy emission is dominated by a sharp peak at around 250 nm. Based on the experimental results and theoretical calculations, the emissive behavior was interpreted according to the mechanism reported in Figure 9. The anti-Kasha emission is attributed to the presence of a T_n_ level easily populated through ISC from the high-energy singlet level (S_5_). Subsequent ISC to S_2_ results in the high-energy long-lived fluorescence. It should be noted that, for this system, a mixed DFT/TDDFT computational protocol has been adopted, where the S_0_ and S_1_ geometries were optimized at B3LYP/6-31G(d,p) level while the higher-energy singlet levels and the triplet states were calculated at M062X/6-31G(d,p) level on the optimized geometry of S_1_.

**Scheme 13:**
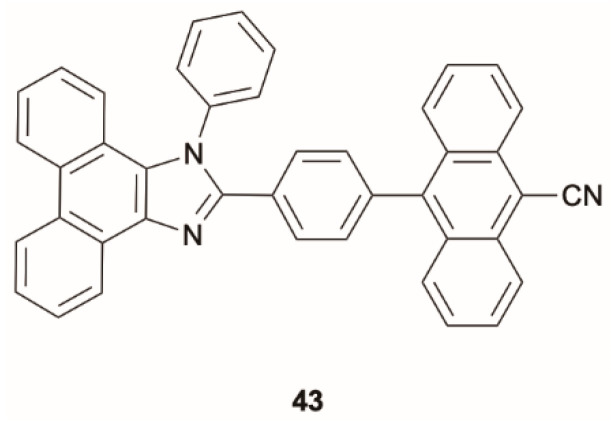
Chemical structure of compound **43**.

**Figure 10:**
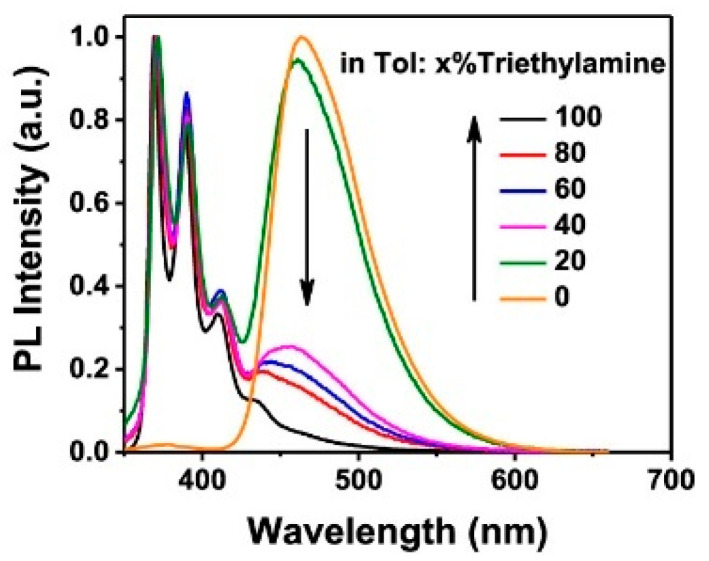
The normalized PL spectra of **43** in toluene and triethylamine mixed solution with different triethylamine fractions. Adapted with permission from [54], Copyright 2019, American Chemical Society.

**Figure 11:**
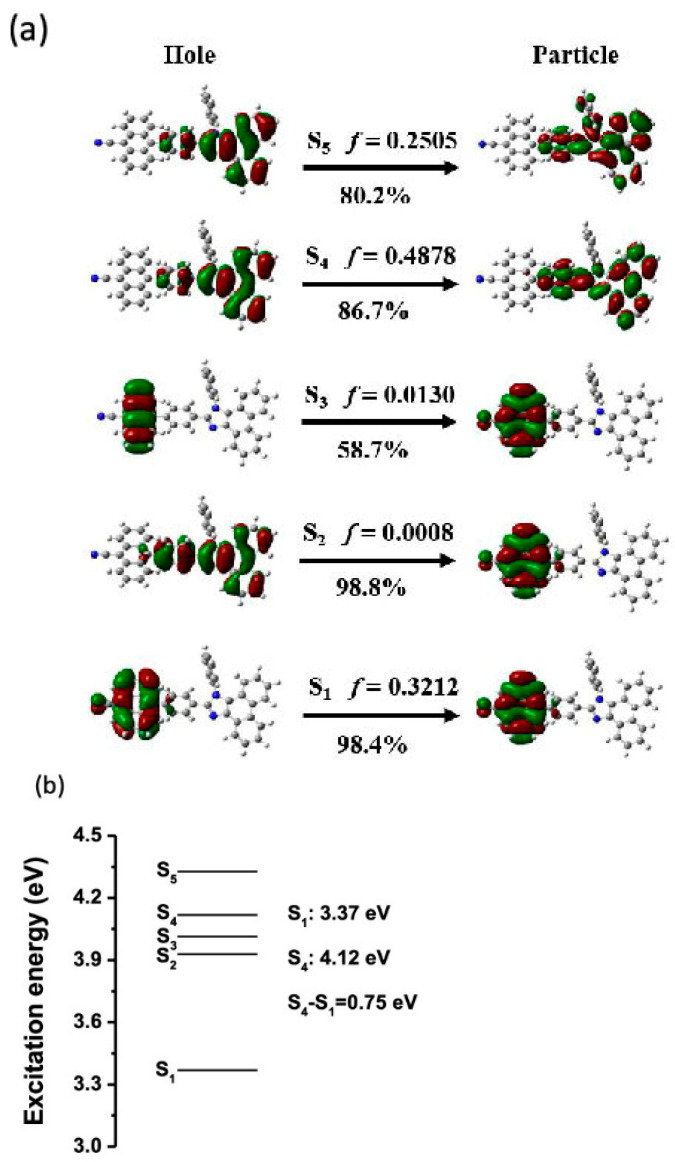
(**a**) Natural transition orbitals of **43**; (**b**) the energy landscape for singlet states of **43**. Reprinted/adapted with permission from [54], Copyright 2019, American Chemical Society.

Jiang et al. [54] reported the photophysical investigation of compound **43** (Scheme 13) in which **PPI** is connected to the anthracene-9-carbonitrile (AnCN) fragment. The absorption bands of the compound are a combination of the independent absorption bands of the **PPI** and AnCN units while the emission displays a solvatochromic behavior, consistent with its CT character. This was supported by DFT calculations which revealed that HOMO and LUMO are delocalized on the **PPI** and AnCN units, respectively, and are almost totally separated. PL spectra in toluene solution display an emission at 463 nm and a very weak one in the 360–400 nm range which becomes predominant when triethylamine is added to the solution (see Figure 10). Through comparative spectroscopical studies, the high-energy emission was associated with the **PPI** unit. Based on TDDFT calculations at the M06-2X/6-31G(d,p) level (see Figure 11), the origin of the two emissions was ascribed to radiative decay from molecular S_4_ and S_1_ excited states. In particular, calculations indicated S_1_ and S_3_ states localized on the AnCN unit, S_2_ state with CT character from **PPI** to AnCN and S_4_ and S_5_ both localized on **PPI** units. Anti-Kasha emission from S_4_ was explained by the poor electronic coupling between S_1_ and S_4_, the high S_4_-S_1_ energy gap (0.75 eV) and the large oscillator strength of S_4_ with respect to S_2_ and S_3_. In agreement with time-resolved analysis, quenching of the low-energy emission by triethylamine was ascribed to exciplex formation between the S_1_ state and triethylamine, which results in an inhibited S_4_-S_1_ IC and therefore in an intensification of the high-energy emission.

## 5. Cyanines

**Figure 12:**
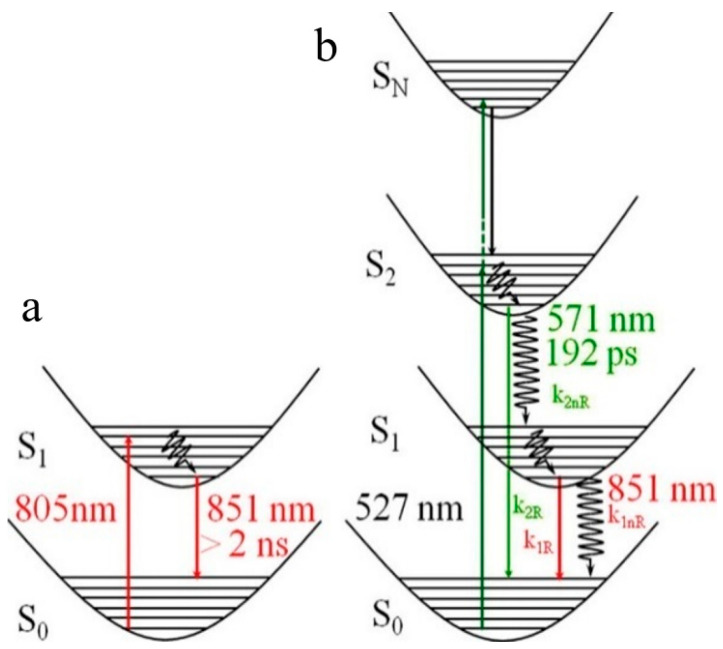
Schematic diagram of excitation of IR125 (**44**) in DMSO by (**a**) 805 nm laser pulse (low-energy excitation) and (**b**) 527 nm laser pulse (high-energy excitation). Incident laser polarization dependent studies have shown the importance of higher excited states in the fluorescence emission as discussed in the text. Reprinted from [55], Copyright 2013, with permission from Elsevier.

**Scheme 14:**
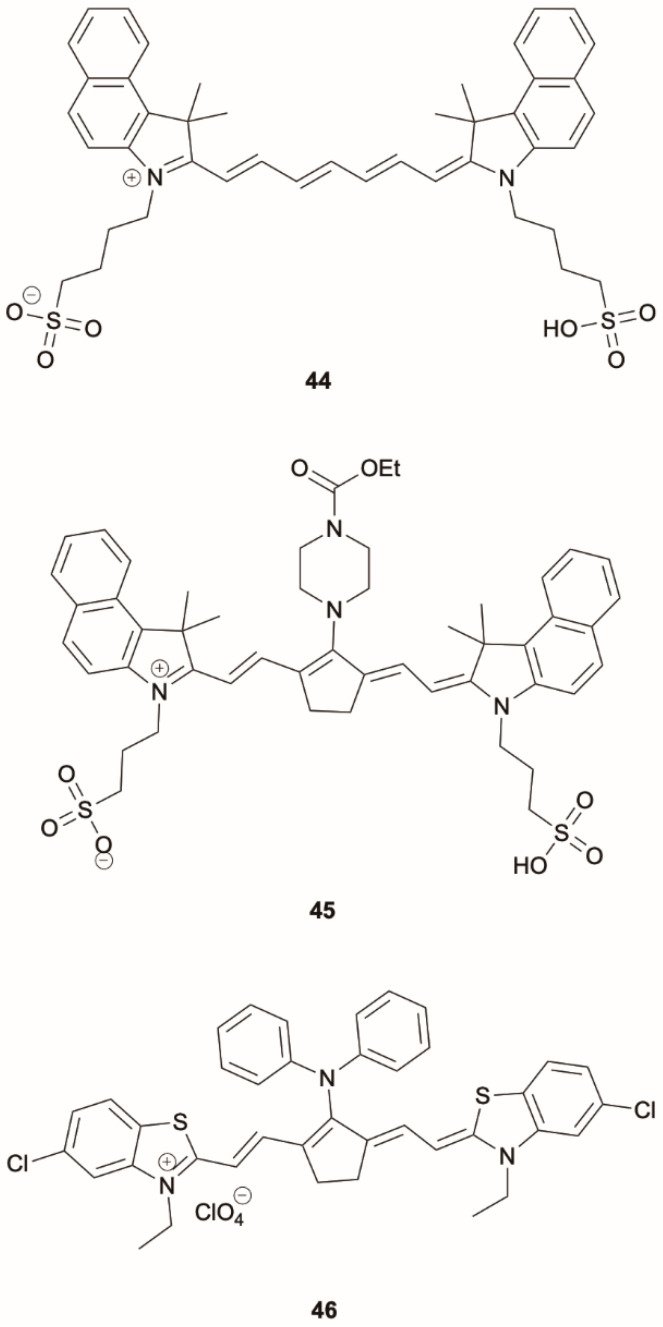
Chemical structure of compounds **44**–**46**.

The peculiar electronic structure of cyanines, having an odd number of conjugated p_z_ orbitals and an even number of π electrons, results in the stabilization of the S_1_ state with respect to S_2_. Therefore, frequently, these dyes are characterized by an intense S_0_-S_1_ transition in the visible/near-IR region and much less intense S_0_-S_n_ (*n* > 1) transitions in the visible/near-UV region. Another relevant aspect of cyanine dyes is their large two-photon absorption cross-sections for near-IR wavelengths. These peculiar properties of cyanine dyes have attracted much interest in many applications such as two-photon photosensitizer or antenna systems. In addition, the large S_2_-S_1_ energy gaps (0.6–1.0 eV) associated with low S_2_-S_1_ IC rates represent an interesting feature for possible anti-Kasha behavior. In fact, some cyanine dyes exhibit dual fluorescence when excited directly to S_2_, as reported by Das et al. [55] in 2013. In particular, cyanine IR125 (**44**) (Scheme 14) in DMSO produces a single fluorescence at 851 nm when excited at 805 nm, but a second fluorescence at 571 nm (of S_2_-S_0_ origin) is activated together with the one at 851 nm (of S_1_-S_0_ nature) upon excitation at 527 nm. According to steady-state and time-resolved experiments, dual fluorescence was schematized as reported in Figure 12. By high-energy excitation, direct fluorescence from S_2_ is produced together with IC to S_1_, from which fluorescence is also observed. By low-energy excitation, only S_1_ is populated, therefore resulting in single fluorescence. 

Later on, Das et al. [56] reported two near-infrared (NIR) tricarbocyanine dyes, IR144 and IR140 (**45** and **46**, respectively) (Scheme 14), displaying two distinct fluorescent emissions in different alcoholic solutions (MeOH, EtOH, PrOH and BuOH): one in the visible region (500–550 nm), assigned to an S_2_-S_0_ transition, and the other in the NIR (850–900 nm), assigned to an S_1_-S_0_ transition. For both **45** and **46**, a variation of the S_2_ emission intensity and lifetimes by varying the alcohol chain length was observed. Differently from **46**, **45** displays an S_2_ excited state dynamics which depends on the solvent polarizability as predicted by the energy gap law. By increasing the polarizability of the alcohol series (from MeOH to BuOH), S_2_ emission lifetimes of **45** can be extended from a few picoseconds to quite larger values (83.46 ps in BuOH), opening up a new area for cell imaging and tissue engineering in different alcohols.

**Scheme 15:**
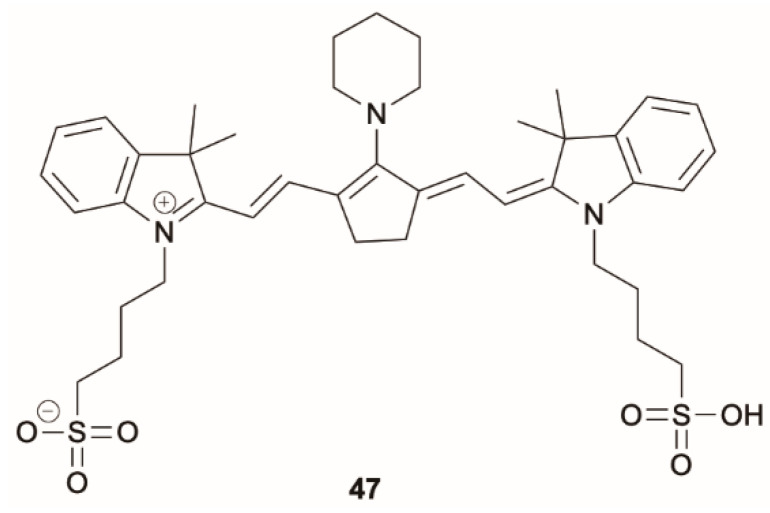
Chemical structure of compound **47**.

Nairat et al. [57] proposed an original way to modulate the Kasha rule through photonic control in cyanine dyes by using shaped pulses for direct S_2_ excitation. The authors tracked the S_2_ and S_1_ fluorescence yield for different cyanine dyes, i.e., IR144 (**45**) and mPi-IR806 (**47**) (Scheme 15), in solution as a function of the femtosecond pulse produced by a linear chirp device. These findings offer a photonic method to control S_2_ population, allowing the exploration of photochemical processes initiated from higher excited states.

**Scheme 16:**
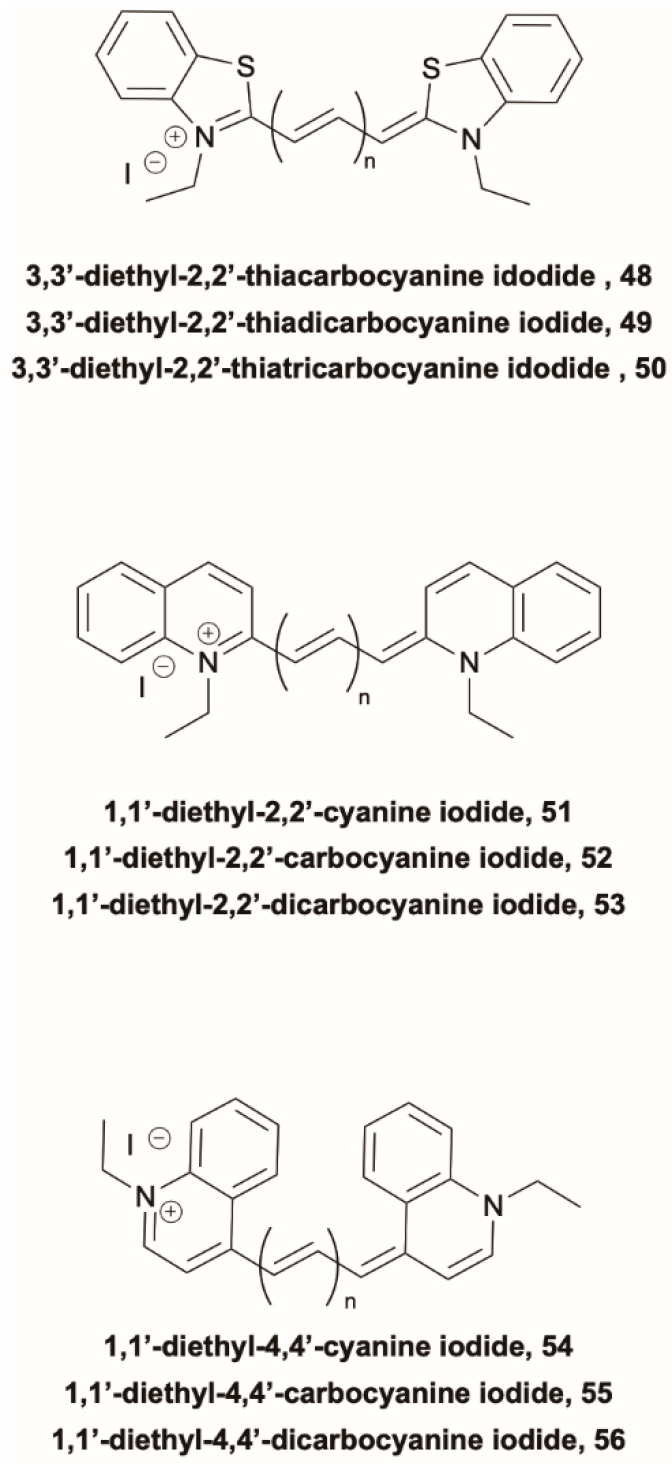
Chemical structure of compounds **48**–**56**.

Guarin et al. [58] reported direct measurements of the S_2_ lifetime for three cyanine families with benzothiazolyl (**48**–**50**) (Scheme 16), 2-quinolyl (**51**–**53**) (Scheme 16) and 4-quinolyl (**54**–**56**) (Scheme 16) end groups and different –(C=C)_n_– skeleton sizes (*n* = 1, 2 and 3 for each family) in order to investigate the structural features able to slow down or inhibit S_2_-S_1_ IC. Clear emission peaks in the region between the first and second absorption transitions, ascribable to emission from S_2_, were detected for the longer cyanines in ethanol solution (at 429 and 494 nm for **49** and **50**, respectively; at about 460 and 490 nm for **52** and **53**, respectively; at 460 and 523 nm for **55** and **56**, respectively), while the shortest ones displayed weak emission from S_2_ only in more concentrated solutions. The dynamics of the upper states was followed by femtosecond fluorescence upconversion and by time-resolving spontaneous emission. Moreover, the time evolution of the S_1_ emission allowed the monitoring of the population growth of S_1_ through IC from S_2_. The authors observed that S_2_ lifetime can vary by more than two orders of magnitude depending on the conjugation length and end groups. For the shortest cyanines, **51** and **54**, ultrafast (<0.4 ps) S_2_ lifetimes were measured, with a correspondingly fast S_1_ population. These rapid dynamics were associated with the nonplanar ground state geometry due to steric hindrance between the end groups. For all the other cyanines (**48**–**50**, **52**, **53**, **55** and **56**), having planar structures, the S_2_ lifetimes were found to closely follow the energy gap law, so that the cyanine with the largest gap, **50**, displays the slowest (17.3 ps in ethanol) S_2_ decay time among all the analyzed compounds.

**Scheme 17:**
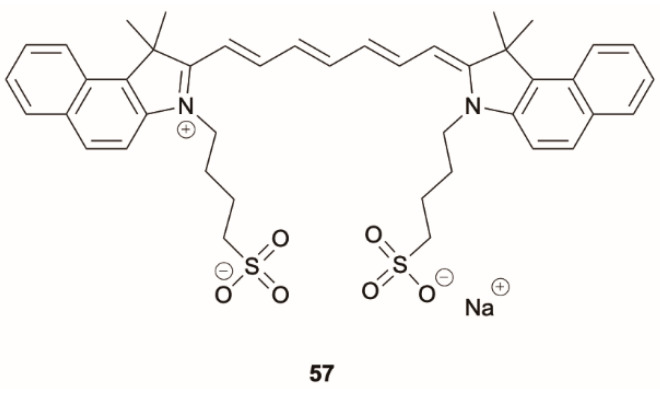
Chemical structure of compound **57**.

Similarly, Kumari and Gupta [59] showed that indocyanine green ICG, **57** (Scheme 17), an exogenous contrast agent used for clinical applications in the NIR region for its 810 nm emission from S_1_, displays direct fluorescence at 572 nm from S_2_ when excited at 393 nm. S_2_ level can be populated also by two-photon excitation with a 790 nm wavelength femtosecond laser, in the well-known “tissue-optical window”, providing a two-photon contrast agent for biomedical imaging applications. The authors demonstrated the use of **57** as exogenous contrast agent for HeLa cell imaging, exploiting its direct emission from the S_2_ state.

## 6. Thiophenes

Thiophene-based compounds are potential candidates to display anti-Kasha phosphorescence from T_2_ since thienyl substituents have been reported, through a combined spectroscopic and B3LYP/6-31G(d) DFT and TDDFT investigation, to favor S_1_ to energy-matched T_2_ ISC [60].

**Scheme 18:**
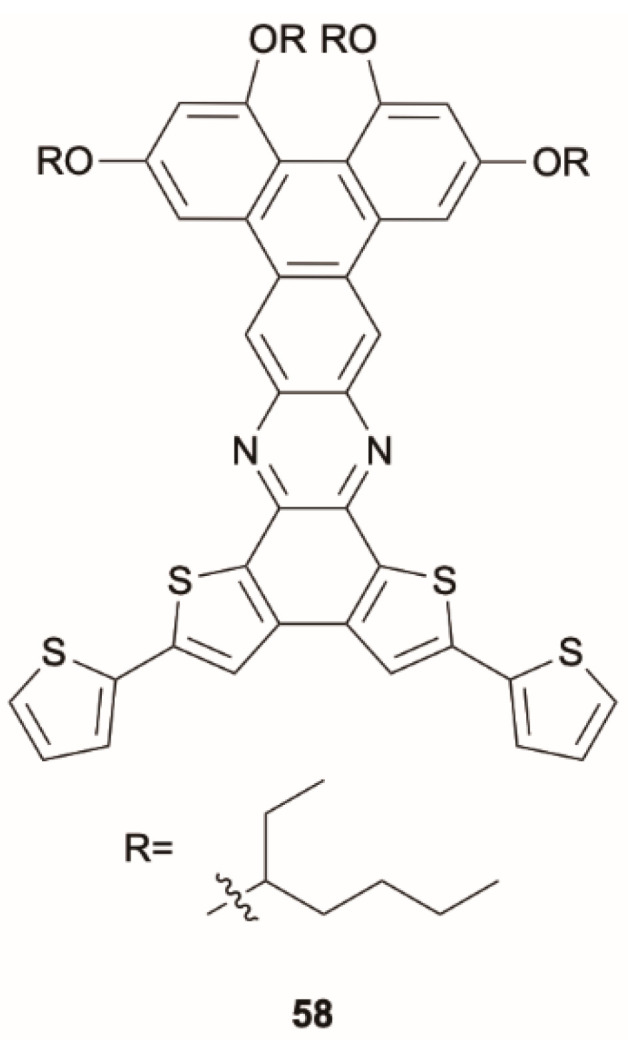
Chemical structure of compound **58**.

**Figure 13:**
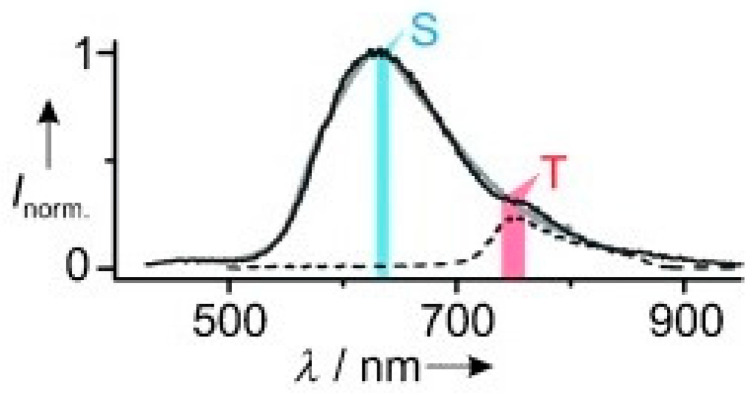
OLED EL spectra of **58** compared to solution PL (gray line) and delayed PL from dilute dispersions in polystyrene films (dashed line). The delayed PL is measured in vacuo to prevent triplet quenching. Adapted with permission from [61].

**Figure 14:**
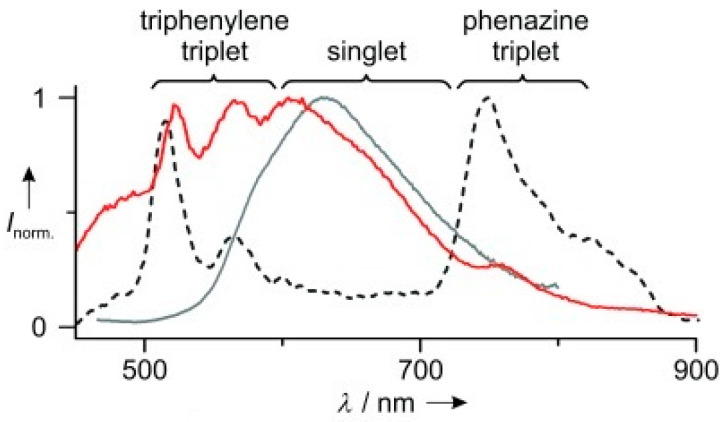
Electrically and optically excited phosphorescence from high-lying triplets of **58**. EL spectrum at 4 K (red), room-temperature solution PL (gray) and gated dilute-film PL (25 K, dashed). Two dominant distinct phosphorescence peaks are identified. Adapted with permission from [61].

Chaudhuri et al. [61] prepared and studied compound **58** (Scheme 18), which has a thiophene-decorated phenazine linked to a triphenylene block. Compound **58** displays in solution one fluorescent emission at 630 nm corresponding to deactivation from S_1_. Concomitant phosphorescent emission at 760 nm at room temperature becomes visible only in polystyrene matrix with exclusion of oxygen (see Figure 13). Intriguingly, room temperature EL spectra of **58** in poly(9-vinylcarbazole) matrix display fluorescence/phosphorescence dual EL (see Figure 14). Moreover, EL and gated spectra of diluted polystyrene films, collected at 4 and 25 K, respectively, show an additional peak at 530 nm from a high-energy triplet. Based on PBE0/6-31G(d) geometry optimization and PBE0/6-311G(d) TDDFT calculations on the analog compound with the long alkyl chains (R) replaced by methyl groups, the two phosphorescences were assigned to triplet states localized on the triphenylene (the high-energy emission) and the phenazine moieties (the low-energy one). At RT, the high-energy triplet emission is quenched by vibrations that favor IC from the “hot” triphenylene triplet to the lower-energy phenazine one. It was therefore suggested that anti-Kasha dual phosphorescence may favor the realization of room-temperature electrophosphorescent devices.

**Scheme 19:**
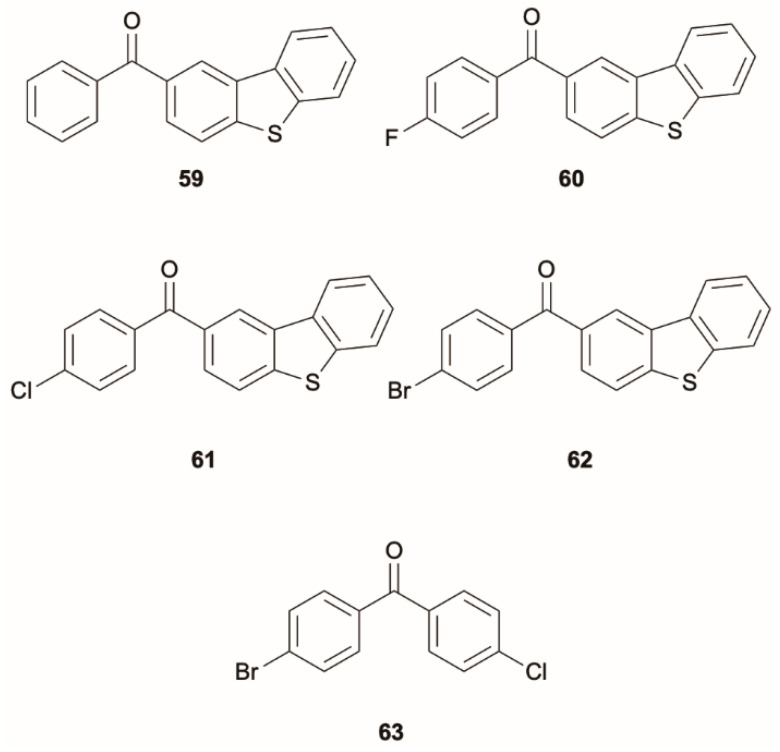
Chemical structure of compounds **59**–**63**.

**Figure 15:**
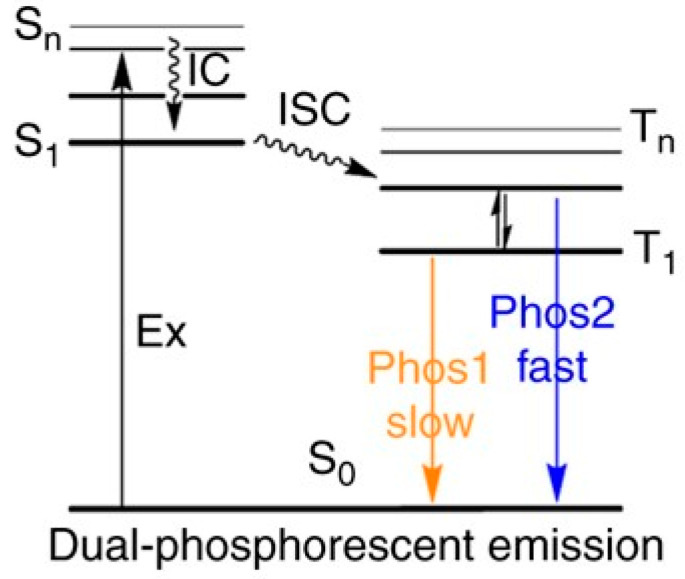
Jablonski diagram for dual phosphorescent emission of **59**–**63**. Adapted with permission from [62]. Copyright 2017, Springer Nature.

He et al. [62] proposed a design strategy to develop pure-phosphorescent single-molecule white light emitters by incorporating benzophenone derivatives into the dibenzothiophene unit. By Friedel–Crafts acylation, five compounds, namely dibenzo[*b*,*d*]thiophen-2-yl(phenyl)methanone (**59**), dibenzo[*b*,*d*]thiophen-2-yl(4-fluorophenyl)methanone (**60**), dibenzo[*b*,*d*]thiophen-2-yl(4-chlorophenyl)methanone (**61**), dibenzo[*b*,*d*]thiophen-2-yl(4-bromophenyl)methanone (**62**) and 4-bromo-4′-chlorobenzophenone (**63**) (Scheme 19) were synthesized, for which phosphorescence was expected to be triggered by the presence of both the carbonyl group and the heavy atom. Moreover, the dibenzothiophene moiety should favor dual phosphorescence by providing various triplet excited states with different molecular orbital configurations. The derivatives display CIE behavior showing weak luminescence in solution and intense emission as crystals at ambient conditions arising from two phosphorescent contributions: a fast one at 470 nm (τ = 0.06–0.71 ms), which has been attributed to a radiative decay from T_2_, and a long-lived one at about 570 nm (τ = 104–123 ms) due to emission from T_1_ (see Figure 15). The assignment of the fast decay to a phosphorescence rather than a TADF was supported by low-temperature measurements, revealing an increase in lifetime with a decrease in temperature. Moreover, quantum mechanics/molecular mechanics (QM/MM) studies on selected compounds, where the QM region was treated at B3LYP/6-31G(d) level for both DFT and TDDFT calculations, provided two lowest excited states (T_1_ and T_2_) below S_1_, with large S_1_-T_1_ separation (in the 0.72–0.88 eV range), suggesting that TADF is less likely to occur at RT. The key point for successfully obtaining dual phosphorescence relies on the phenyl group shared between benzophenone and dibenzothiophene moieties providing, respectively, (*n*, π*) and (π, π*) character to the transitions. As a result, although both T_1_ and T_2_ states have mixed (*n*, π*) and (π, π*) configuration, T_2_ is mainly an (*n*, π*) transition while T_1_ possesses more (π, π*) character. According to the El-Sayed rule, a larger SOC is expected for T_2_-S_0_ than for T_1_-S_0_. This leads to a short lifetime for T_2_ and a long lifetime for T_1_. The anti-Kasha emission might then occur after the thermal population of T_2_ from T_1_ due to a small ΔE(T_1_-T_2_) (0.19 and 0.27 eV from experiment and theory, respectively, for **61**), comparable to RT thermal energy. 

Later on, Paul et al. [63] applied computational techniques to re-examine the previously reported anti-Kasha behavior of **61** [62]. According to the new calculations, performed by DFT and TDDFT approaches at the Grimme’s dispersion corrected B3LYP/6-311G(d,p) level of theory, the ΔE(T_1_-T_2_), 0.48 eV, is too large to obtain a sufficient Boltzmann population of T_2_ from T_1_. Therefore, they proposed that T_2_ is instead populated by S_1_-T_2_ ISC. These two levels are in fact separated by only 0.09 eV and are characterized by strong Duschinsky mixing [64] between their normal modes. 

**Scheme 20:**
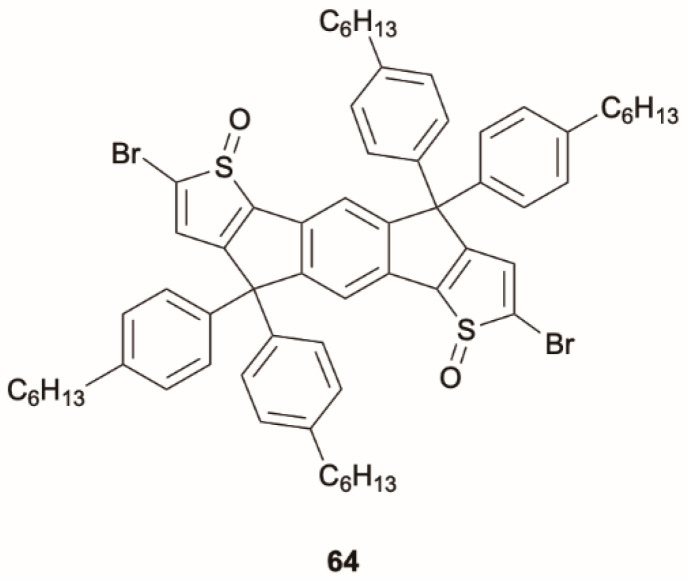
Chemical structure of compound **64**.

Cao et al. [65] prepared and investigated a series of highly planar indaceno[1,2-*b*:5,6-*b*′]dithiophene 1,6-dioxide derivatives containing out-of-plane side chains aimed at preventing quenching of the emission due to strong intermolecular π–π interactions. Among the studied compounds, film of **64** (Scheme 20) displays anti-Kasha phosphorescence.

**Figure 16:**
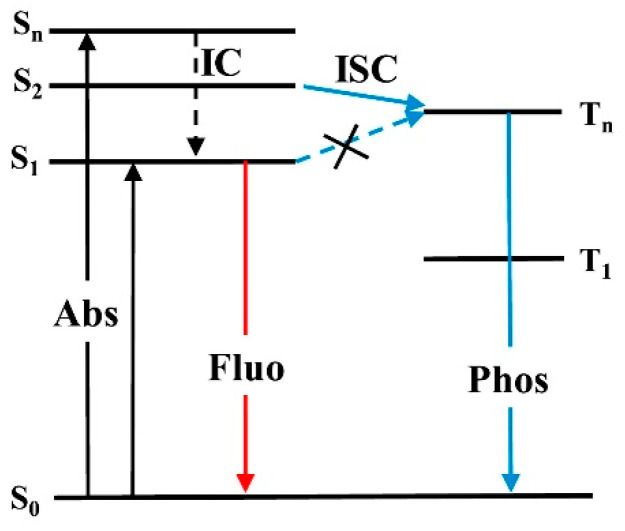
The proposed mechanism for the anti-Kasha properties of **64** film. Reprinted from [65], Copyright 2020, with permission from Elsevier.

Diluted chloroform solution of **64** displays, when excited at 380 nm, a vibrationally resolved emission originating from a locally excited state at 400–500 nm and an additional broad band of ICT character at 500–700 nm. The film of **64** shows excitation-dependent photoluminescence. In particular, upon excitation at 340 nm, a fluorescent band at 570 nm appears in the spectrum. While, at 253 nm excitation, dual emission comprising a phosphorescence at 400 nm (τ = 0.102 ms) and a fluorescence at 570 nm (τ = 0.812 ns) is observed. The phosphorescence was attributed to anti-Kasha deactivation originating from a high-energy triplet level. In agreement, DFT-TDDFT calculations on a model molecule where hexyl was replaced by methyl, performed at B3LYP/6-31G(d) level for S_0_ and S_1_ and at M06-2X/6-31G(d) level for higher singlet states and for triplet states, revealed small S_2–3_-T_3–4_ energy gaps and a large T_3_-T_2_ one. This justifies an easy ISC to populate high triplet levels from which phosphorescence is observed due to inhibited IC. Therefore, it was suggested that upon excitation to S_1_, only the corresponding fluorescence at 570 nm is observed. However, by populating upper excited states (S_n_), relaxed S_1_ (by IC) and T_n_ (by ISC) are obtained, from which fluorescence and anti-Kasha phosphorescence are respectively produced (see Figure 16). 

## 7. Carbazoles

**Scheme 21:**
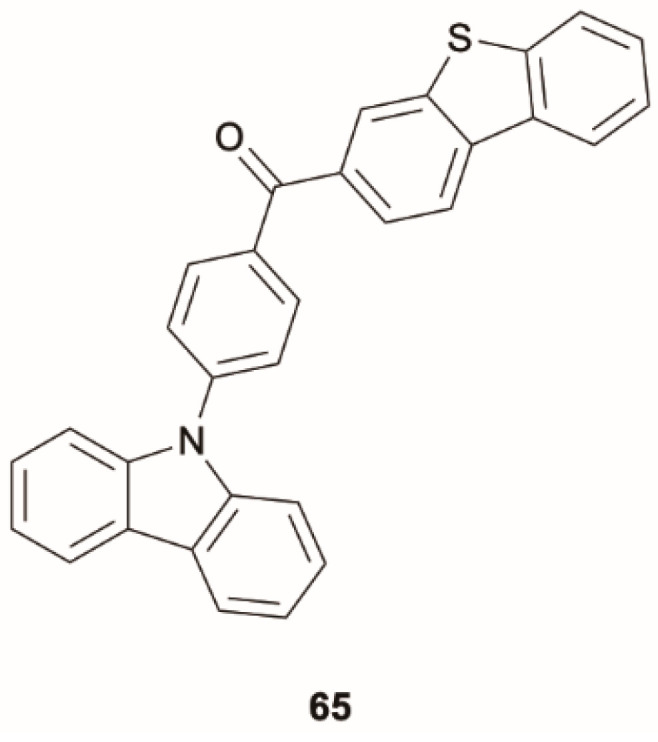
Chemical structure of compound **65**.

Carbazole possesses a high triplet energy level which induces a small energy gap between triplet and singlet states beneficial for ISC [66]. Feng et al. [67] developed a fully organic compound, **65** (Scheme 21) (containing carbazole (Cz), a carbonyl group (Cb) and dibenzothiophene (DBT)), able to display in DCM solution RTP from T_2_ (at 485 nm) with Φ varying from 25% in air to 40% under nitrogen. The ICT features of such emission were proven by a significant solvatochromic red shift with increasing solvent polarity. Experimental studies performed at 77 K revealed for the molecule three radiative deactivation paths assigned to S_1_, T_2_ and T_1_, with bands respectively at 440, 465 and 570 nm, originating from the same photogenerated excited state. Based on the observation that, among the three bands, that associated with T_2_ is the strongest while that originating from T_1_ is the weakest and has the longest lifetimes, an easy S_1_-T_2_ ISC and a slow T_2_-T_1_ IC were suggested. Such interpretation was supported by B3LYP/6-31G(d) DFT and TDDFT calculations in DCM, which provided two lowest triplet excited states, T_1_ and T_2_, both of mixed (*n*, π*) and (π, π*) character, below the (π, π*) S_1_ level, with ΔE_ST_ energy gaps equal to 0.305 and 0.04 eV, respectively. While T_1_, mainly localized on the Cz unit, has a predominant (π, π*) contribution, T_2_ is distributed on the CbDBT moiety and is principally (*n*, π*) in nature. Moreover, the computed SOC constant, ξ_ST_, is larger for T_2_ (4.69 cm^−1^) than for T_1_ (1.11 cm^−1^). Therefore, theoretical results confirm that, on one side, S_1_-T_2_ ISC is facilitated with respect to the S_1_-T_1_ one and, on the other side, T_2_-T_1_ IC is inhibited owing to spatial separation, altogether explaining the observed anti-Kasha emission from T_2_.

**Scheme 22:**
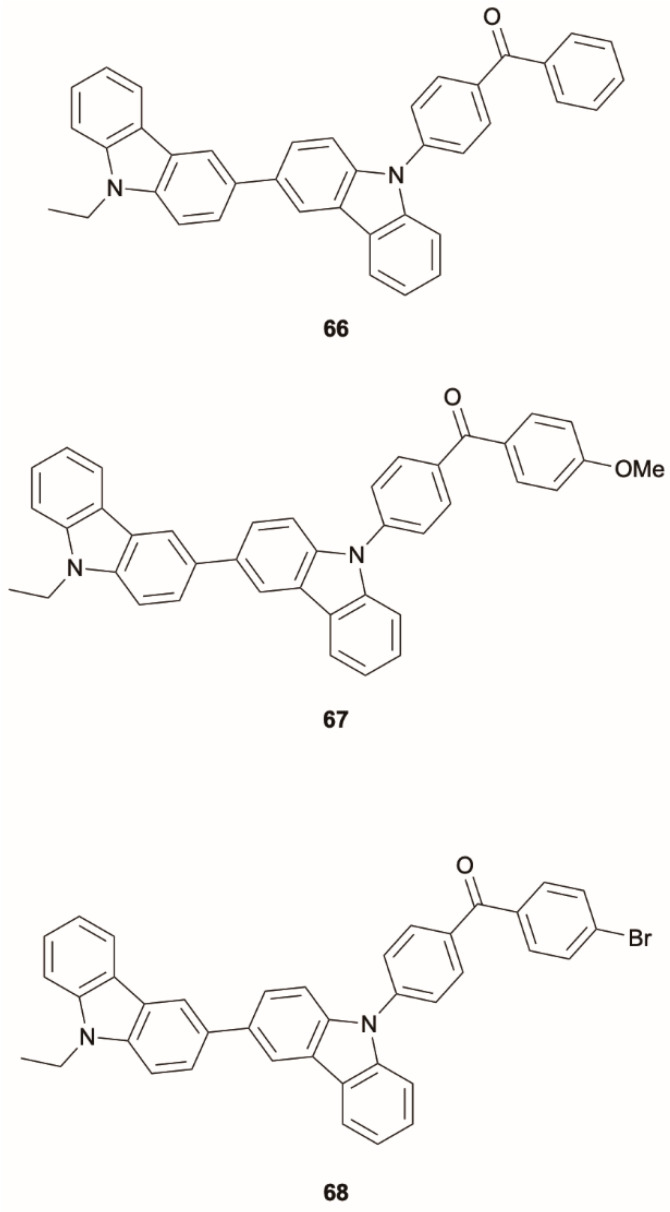
Chemical structure of compounds **66**–**68**.

Wang et al. [68] prepared and fully photophysically characterized both in solution and in the solid state three 4,4′-biscarbazole derivatives, **66**–**68** (Scheme 22).

The three compounds are not emissive (**67** and **68**) or are hardly emissive (**66**) in diluted THF or acetone solutions at RT and exhibit a fluorescence (in the 410–430 nm range) and a long-lived component (in the 460–490 nm range) at 77 K. In solvent/nonsolvent (water) mixtures, AIE long-lived (≈3 μs) features become visible at RT. Powders of **66**–**68** at RT do not display fluorescent emissions but exhibit long-lived components with a lifetime in the microsecond range (Φ = 35–64%) whose dual phosphorescence origin was established by steady-state and time-resolved experiment at different temperatures and assigned to T_2_ and T_1_ excited states. Assignment and mechanism of the observed dual phosphorescence were supported by TDDFT calculations performed at the ωB97XD/6-311G(d) level of theory. For **66**, calculations revealed the presence of three triplet excited states, T_1_ and degenerate T_2_ and T_3_, below S_1_. The four excited states possess mixed (*n*, π*)/(π, π*) character, and, being S_1_ and T_1_ of prevailing (*n*, π*) and (π, π*) contribution, respectively, an easy ISC from S_1_ to T_1_ can be predicted based on El-Sayed rules. Similarly, T_2_, despite its dominant (*n*, π*) character, can be easily populated from S_1_ through degenerate T_3_ of main (π, π*) nature. Radiative deactivation from T_2_ competes with IC to T_1_ due to the relatively large T_1_-T_2_ energy gap. Similar results were obtained for **67** and **68**, for which an S_1_ of predominant (*n*, π*) contribution and T_1_ and T_2_ dominated by a (π, π*) one were determined. The potentiality of the RTP features of the compounds was investigated, except for **68** having the lower quantum efficiency, in nondoped OLED devices based on their homogeneous thin films. TmPyPB, m-MTDATA (4,4′,4″-Tris[(3-methylphenyl)phenylamino]triphenylamine) and TAPC were used as electron-transporting, hole-injecting and hole-transporting layers, respectively. The **67** device displays a relatively small efficiency roll-off of about 20% at 1000 cd/m^2^ with a luminance up to 4019 cd/m^2^. Moreover, thanks to the combination of AIE and RTP characteristics, the η_ext_ of device **66** reached beyond the theoretical η_ext_ limit of fluorescence-based OLED.

## 8. Miscellaneous

**Scheme 23:**
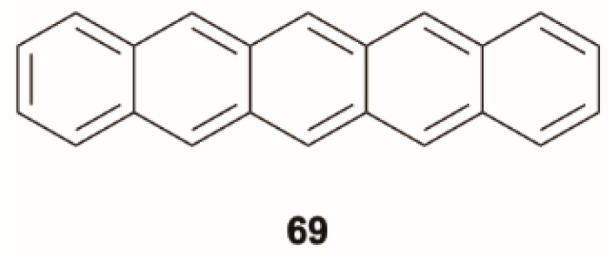
Chemical structure of compound **69**.

**Figure 17:**
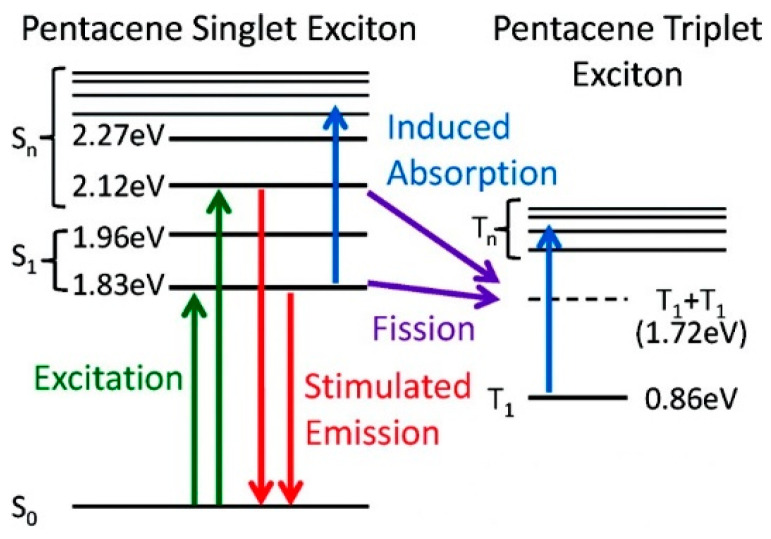
Energy level diagram for excitonic states in polycrystalline thin films of **69**. The labeled arrows indicate the main transitions. The energy position of the Davydov levels of the first singlet and higher-lying exciton are reported. Reprinted with permission from [69], Copyright 2011, American Chemical Society.

Wilson et al. [69] probed the process of exciton fission in polycrystalline thin films of pentacene, **69** (Scheme 23), by ultrafast TA spectroscopy. The authors monitored the singlet population through the detection of stimulated emissions (SEs), which were found to be excitation-dependent. The anti-Kasha blue-shifted SE obtained by excitation of higher-lying transitions was attributed to light emitted by the “hot” exciton before its relaxation to S_1_ (as suggested by the red arrows in Figure 17). The dynamics of triplet generation indicated that the singlet fission process does not require first relaxation to S_1_ but can proceed directly from higher-lying singlet states on a similarly rapid (~80 fs) time scale. These observations imply that pentacene S_1_ state is short-lived, even on the time scale of excitonic cooling, in agreement with the very low luminescence efficiency of pentacene crystals.

**Scheme 24:**
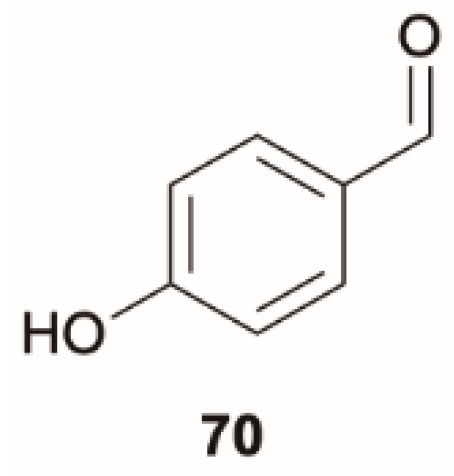
Chemical structure of compound **70**.

**Figure 18:**
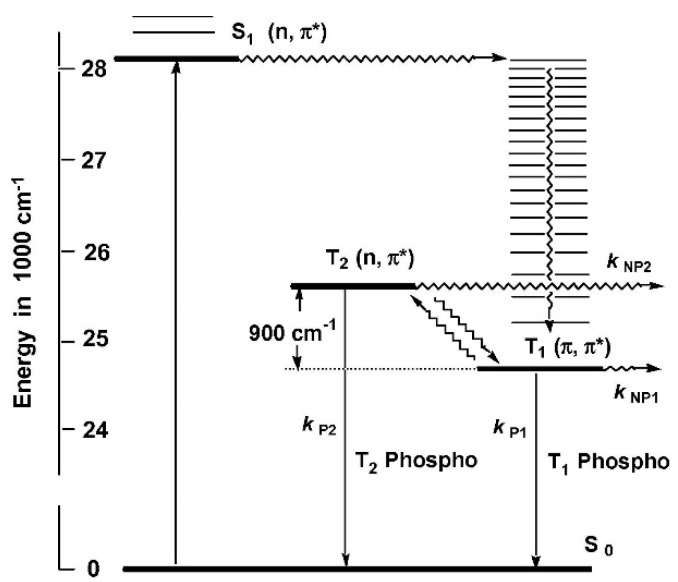
Energy level scheme showing the relaxation processes for **70** in a *p*-dichlorobenzene matrix: k_P1_ and k_NP1_ are, respectively, the radiative and nonradiative rate constants of T_1_; k_P2_ and k_NP2_ are those of T_2_. Reprinted from [70], Copyright 2014, with permission from Elsevier.

Some derivatives of benzaldehyde [9] were reported to emit from T_2_ due to its closeness to T_1_ and to the higher S_0_-T_2_ oscillator strength with respect to the S_0_-T_1_ one. However, for hydroxybenzaldehyde, **70** (Scheme 24), despite the S_1_ (*n*, π*) > T_2_ (*n*, π*) > T_1_ (π, π*) excited-state ordering being favorable to observing phosphorescence from T_2_, only phosphorescence from T_1_ was observed in a rigid matrix at 77 K [9]. In 2014, Itoh [70], speculating that anti-Kasha emission from T_2_ could be missed due to its weakness, performed a detailed steady-state and time-resolved investigation of **70** in a 1,4-dichlorobenzene matrix in the −196 to −20 °C interval. At 77 K the only phosphorescent emission at 405 nm was assigned to the T_1_ (π, π*) state. At −70 °C an additional phosphorescent contribution at 391 nm of T_2_ (*n*, π*) origin, with intensity increasing with temperature up to −20 °C, was observed. A ΔE(T_1_-T_2_) equal to 0.11 eV was estimated from both steady-state and time-resolved experiments which allowed the drawing of the mechanism reported in Figure 18 in which the thermal population of T_2_ from T_1_ justifies the observed emissive behavior.

**Scheme 25:**
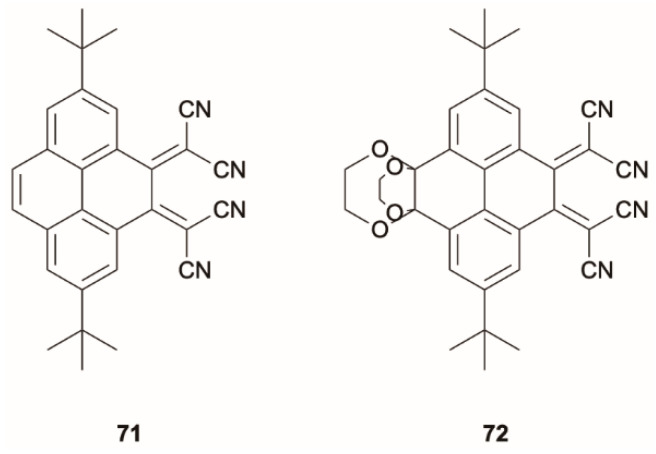
Chemical structure of compounds **71** and **72**.

**Figure 19:**
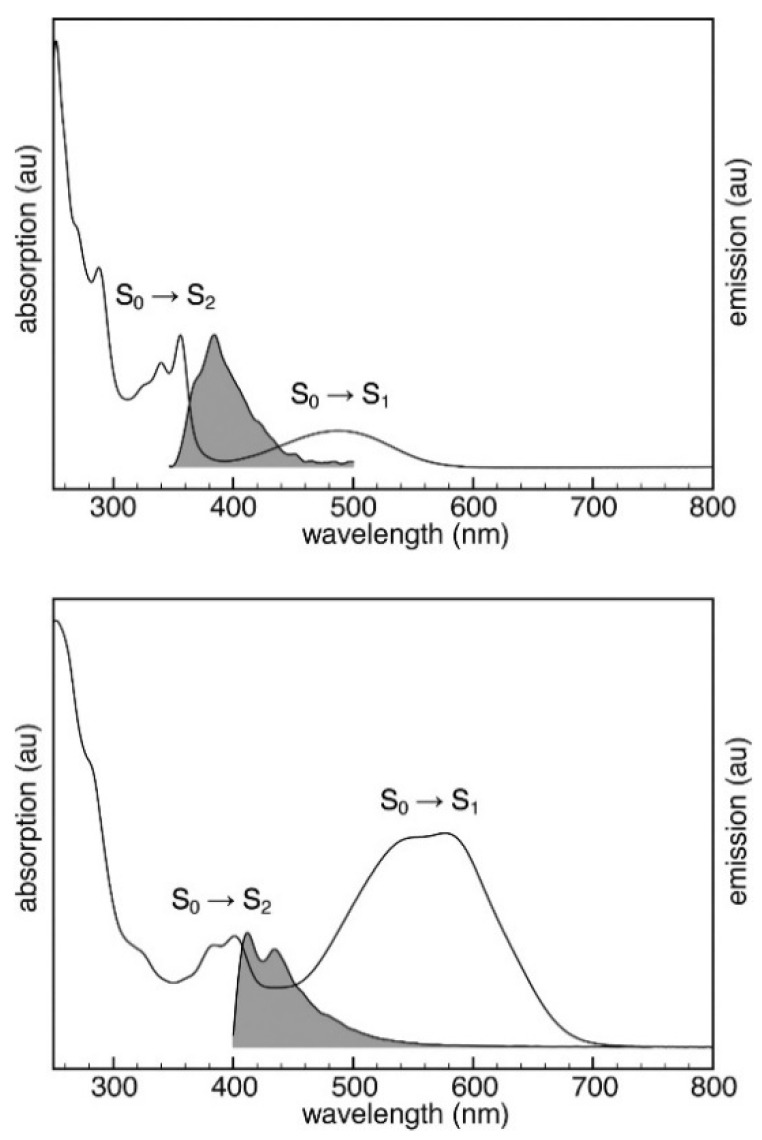
Absorption (white) and emission (gray) features for **71** (**top**) and **72** (**bottom**) in DCM. Reprinted with permission from [71], Copyright 2014, American Chemical Society.

While pyrene, previously reported as an exception to Kasha rule [9], was recently demonstrated [14] to emit uniquely from S_1_, the introduction of dicyano methane substituents on the pyrene scaffold to generate compounds **71** and **72** (Scheme 25) promotes, in DCM solution, an intense ICT emission that originates from S_2_, while no radiative deactivation is obtained upon S_0_-S_1_ excitation [71] (see Figure 19). Violation of the Kasha rule was attributed to the large S_1_-S_2_ energy gap (0.98 and 0.94 eV in **71** and **72**, respectively, as determined by DFT and TDDFT calculations at B3LYP/6-31G(d,p) level) favoring emission from S_2_ over S_2_-S_1_ IC as a consequence of the reduced overlap between vibrational levels of S_1_ and S_2_.

**Scheme 26:**
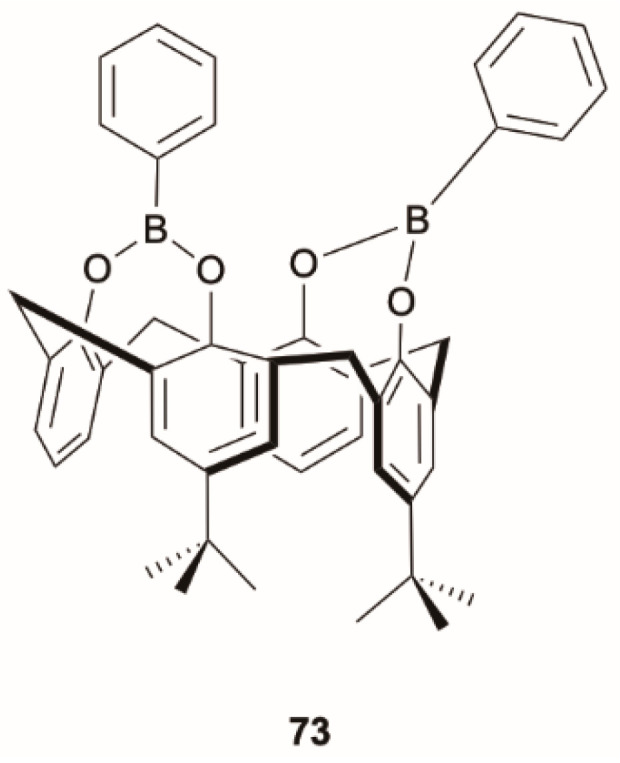
Chemical structure of compound **73**.

Bis(bora)calix[4]arene, **73** (Scheme 26), was reported by Arimori et al. [72] as a fluorescent sensor selective for fluoride due to a decrease in UV absorption and fluorescence upon F^−^ binding. In 2016, Jin et al. [73] performed a thorough DFT/TDDFT study to interpret this behavior. Calculations were performed by using a GGA functional, PBE1W, which provided a better agreement with experimental data, and the 6-311G(d) basis set. The prompt fluorescence of **73** was assigned to emission from S_2_ owing to a sizable ΔE(S_1_-S_2_) gap (0.48 eV) and very low S_0_-S_1_ oscillator strength. The quenching of fluorescence observed for the fluoride-binding complex **73**-F was explained by a theoretically predicted fast IC to a dark S_1_ state.

**Scheme 27:**
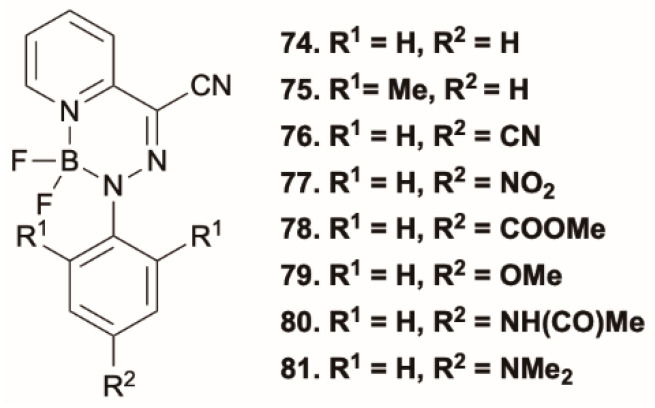
Chemical structure of compounds **74**–**81**.

Qian et al. [74] reported on a series of boron difluorohydrazone derivatives, **74**–**81** (Scheme 27), showing enhanced emission in the solid state and in highly viscous environments and a quenched emission in nonviscous solvents. Based on DFT/TDDFT calculations at the PBE/TZVP level, the authors attributed this behavior to the presence, in these molecular rotors, of an S_1_ dark state and of a higher excited state, S_n_ (with *n* > 1, varying within the series of compounds), having high oscillator strength. In nonviscous solvents, the free intramolecular rotation in the excited state leads to a conformation having a low S_n_-S_1_ energy gap, which allows rapid S_n_-S_1_ IC and then very weak emission or nonradiative decay to S_0_. Highly viscous solvents, on the other hand, inhibit the formation of the nonradiative excited-state structure, decreasing the rate of S_n_-S_1_ IC and then resulting in anti-Kasha emission from S_n_. Such behavior was supported by a strict correlation between the viscosity sensitivity of the fluorescence and the computed barrier to rotation in the excited state: the higher the barrier, the greater the viscosity sensitivity. Moreover, the anti-Kasha emission was experimentally confirmed in **81** by the observation of different absorbance and circular dichroism band shapes at 77 K, a feature that can be explained by the existence of two or more distinct electronic transitions. Furthermore, a weak emission emerged when excitation was shifted towards lower energies, indicating that the molecule is relaxing from the dark state S_1_.

Subsequently, Zhou et al. [75], contesting the use of the pure functional PBE adopted by Qian et al. [74] for **74**–**81**, performed a new TDDFT study on the same series of compounds using the B3LYP and CAM-B3LYP functionals, besides complete active space self-consistent field (CASSCF) calculations. It was reported that S_1_ for the same series of compounds **74**–**81** is not a dark but a bright state, which calls into question the anti-Kasha origin of the enhanced emission of these compounds in viscous environments. The authors proposed an alternative origin, based on restriction, in viscous solvents, of flip-flop motion promoting fast S_1_-S_0_ IC. Confutation of the previously proposed anti-Kasha behavior of these compounds, however, still deserves experimental validation. 

**Scheme 28:**
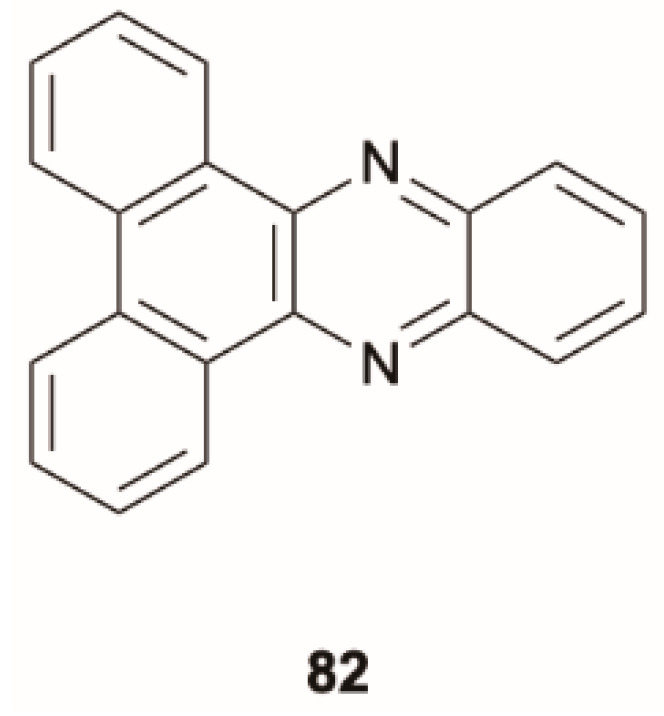
Chemical structure of compound **82**.

**Figure 20:**
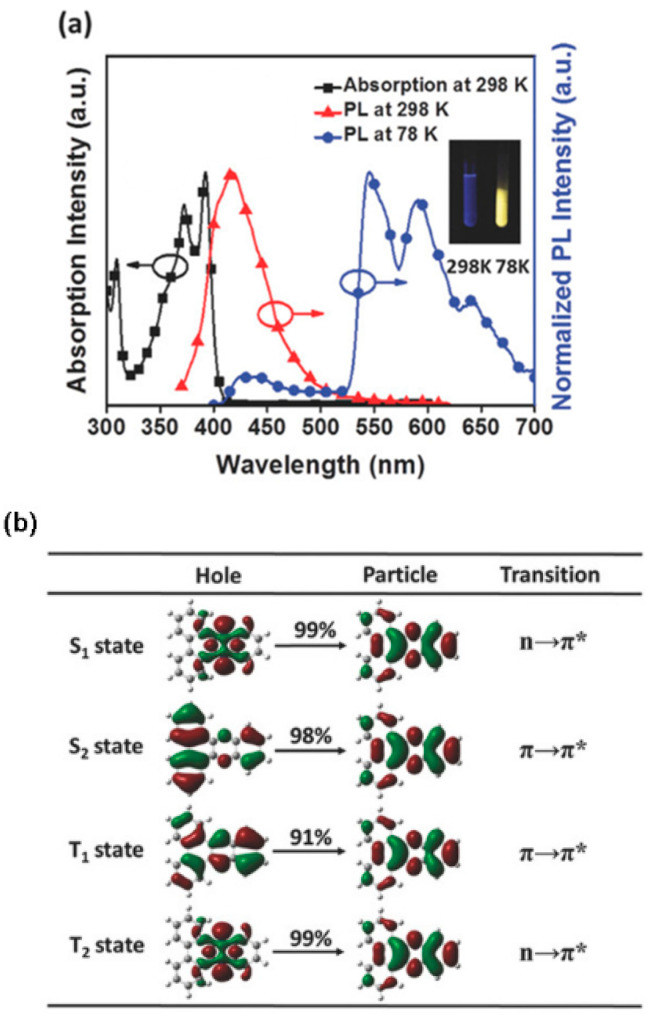

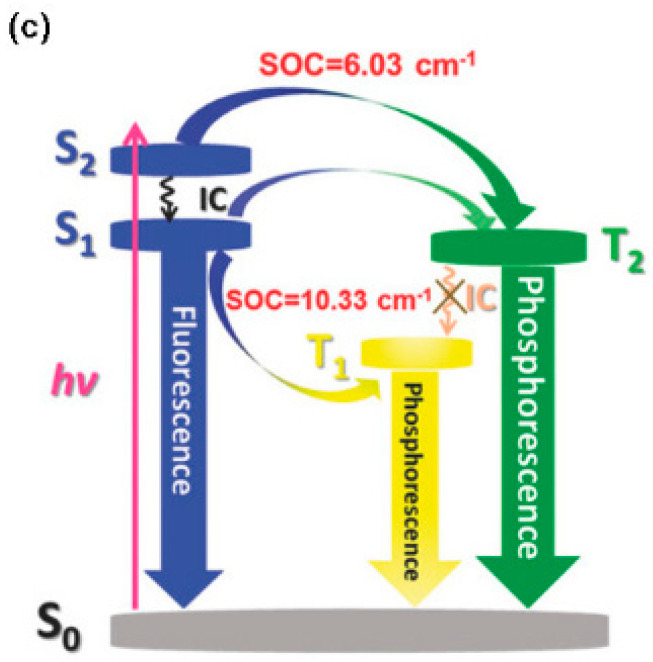
(**a**) Absorption and emission spectra of **82** in THF solution at 298 and 78 K. The insets are the photos of the steady-state emission. The excitation wavelength is 390 nm. (**b**) Natural transition orbital (NTO) images of the S_1_, S_2_, T_1_ and T_2_ excited states of **82**. The NTO images of each excited state represent their transition forms from the “hole” unity to the “particle” unity, respectively, and the percentages on the arrows are the proportions of the transitions. (**c**) Schematic Jablonski diagram with SOC matrix elements of RTP-related ISC processes in **82**. Adapted with permission from [76].

Zhou et al. [76] reported single-molecule white light emission from dibenzo[*a*,*c*]phenazine, **82** (Scheme 28). In solution, **82** displays steady-state PL dominated by a structured phosphorescence with a lifetime of 2.7 μs, accompanied by a fluorescent emission. This latter was associated to an S_1_ state with (*n*, π*) character while the phosphorescence was assigned to a (π, π*) T_1_ level, with S_1_-T_1_ ISC facilitated by El-Sayed considerations (see Figure 20), as confirmed by the very short fluorescence lifetime of **82** (0.07 ns) [77]. Powders of **82** display multiple emissions comprising one S_1_-S_0_ fluorescence and two RTPs, reaching Commission Internationale de l’Éclairage (CIE) coordinates of (0.28, 0.33) and Φ of 1%. From steady-state measurements at variable temperature and time-resolved studies, the authors demonstrate that the two RTPs are relatively independent of each other and excluded a T_1_-T_n_ thermally activated reversed IC. With the support of TDDFT calculations, performed at the B3LYP/6-31G(d,p) level of theory, the high-energy RTP was assigned to a T_2_ state of (*n*, π*) character. T_2_-T_1_ IC competes with T_2_ radiative deactivation, leading to the dual phosphorescent emission. The excitation wavelength dependency of the RTPs demonstrated that the high-energy one could be efficiently generated through multiple alternative ISC channels (S_2_-T_2_ and S_1_-T_2_, see Figure 20c). 

**Scheme 29:**
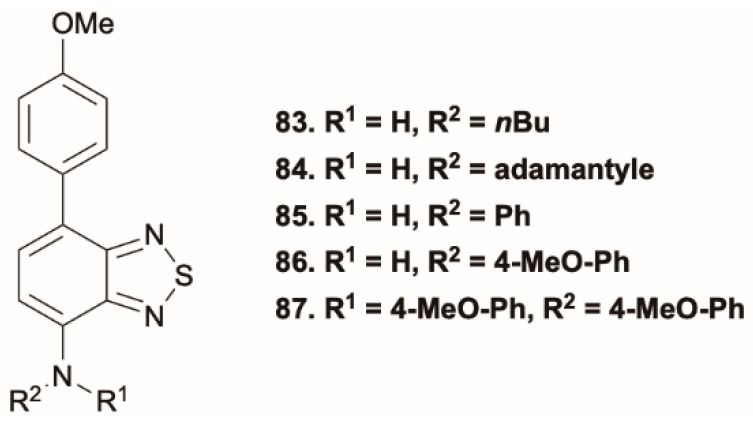
Chemical structure of compounds **83**–**87**.

Peng et al. [78] investigated the photophysical properties in different states of a series of amino benzothiadiazoles. Anti-Kasha dual emission was observed in solution for **83**–**87** (Scheme 29) and was correlated to the strength of the electronic communication between the amino group and the benzothiadiazole moiety. In fact, by increasing the CT character of the molecule, the molecular rigidity is increased and S_2_-S_1_ IC is inhibited. For **83** and **84**, having the strongest CT character and the highest planarity among the members of the family, excitation-dependent dual emission in the visible region was observed both in toluene and acetonitrile solutions. Compounds **85** and **86**, characterized by reduced electron-donating ability from the amino group, display dual emission only in acetonitrile where the ICT is enhanced. For **87**, dual emission appears in both solvents but is hardly visible in acetonitrile. 

**Figure 21:**
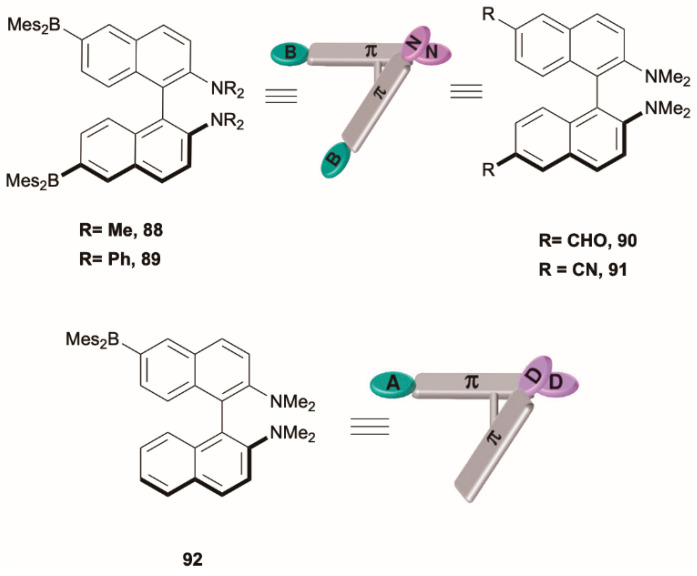
Schematic presentation and structure of *o*,*o*′-binaphthyls **88**–**92** consisting of two D–π–A subunits. Adapted with permission from [80].

Sun et al. [79] and Liu et al. [80] revealed a family of highly twisted *o*,*o*′-substituted binaphthyls, **88**–**92**, featuring a D–π–A structure with ICT character within each aryl group (Figure 21). These compounds, unlike the “normal” D–π–A molecules, consist of two independent D–π–A subunits, each of them bearing either dimesitylboryl, Mes_2_B (**88**, **89**, **92**) or CHO (**90**) or CN (**91**) groups as strong electron acceptors, and NR_2_ groups, with R = Me (**88**, **90**–**92**) or Ph (**89**) as donors. All these compounds revealed temperature-dependent dual fluorescence and switchable circularly polarized luminescence (CPL). Comparison between **88** and **89** [79] indicates that, in the presence of the less bulky NMe_2_ group (**88**), the two fluorescence bands are well separated in polar solvents and are very sensitive to temperature, enabling the use of **88** as a ratiometric fluorescence thermometer. On the other hand, for **89**, the dual emission was observed only in the CPL spectra, probably due to the overlap of the two fluorescences. Combining experimental and theoretical results, the latter obtained by DFT and TDDFT calculations at the PBE0/6-31G(d) level of theory, the dual fluorescence of **88** was assigned to deactivation from S_1_ and S_2_. The computed excitation energies of these two states were very similar (ΔE = 0.07 eV), justifying a thermal equilibrium population between them which explains the observed temperature-dependent dual fluorescence. Moreover, the optimized geometries of S_1_ and S_2_ reveal different features, S_1_ having mainly inter-subunit CT character and S_2_ having mixed intra-subunit CT and LE character. Calculations on **89** provided essentially identical features; owing to its higher structural rigidity with respect to **88**, only small changes are observed in the optimized S_1_ and S_2_ geometries with respect to the ground state. This explains the observed overlap of the S_1_-S_0_ and S_2_-S_0_ deactivations.

From comparative analysis of the effect of the A group in **88**, **90** and **91** [80], it appeared that the acceptor groups greatly affect the relative intensity and the energy separation of the two emissions. The reference compound **92**, lacking one electron-accepting group, was found to possess less sensitive temperature-dependent dual fluorescence, indicating that the symmetric structure of **88**–**91** is very important for realizing highly sensitive temperature-dependent dual fluorescence.

**Scheme 30:**
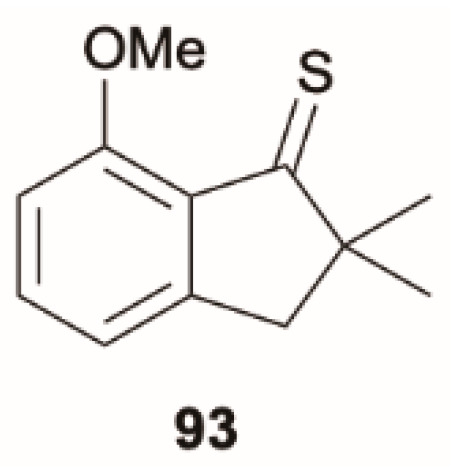
Chemical structure of compound **93**.

**Figure 22:**
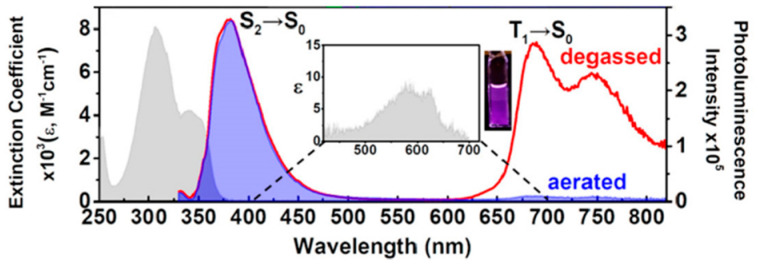
Absorption (gray filled area) and emission spectra (blue filled area and solid red line) of **93** in cyclohexane at room temperature. Insets: expansions of the absorption spectra over specified wavelength regions. λ_ex_: 350 nm. Note that the labeling “aerated” refers to aeration in atmosphere. Adapted with permission from [81], Copyright 2019, American Chemical Society.

Liu et al. [81] reported a study on intramolecular O–H^…^S H-bond formation in a thione derivative which displays dual RTP due to a remarkable photoinduced intramolecular H-bond on/off switching reactions. The corresponding methylated compound, **93** (Scheme 30), which lacks the dual RTP, represents instead an example of anti-Kasha fluorescence (see Figure 22). In cyclohexane at RT, **93** displays dual emission comprising one fluorescence (at 380 nm) and an RTP (at 690 nm). Through a deep photophysical analysis, integrated with DFT and TDDFT calculations at the B3LYP/6-311++G(3df,3pd) level of theory, the authors assigned the first excited state, S_1_, to an optically forbidden (*n*, π*) transition and the higher-lying S_2_ state to an allowed (π, π*) one. The calculated ΔE(S_2_-S_1_) energy gap, amounting to about 1.37 eV, justifies an anomalously slow S_2_-S_1_ IC, allowing the radiative S_2_-S_0_ transition to become competitive.

**Scheme 31:**
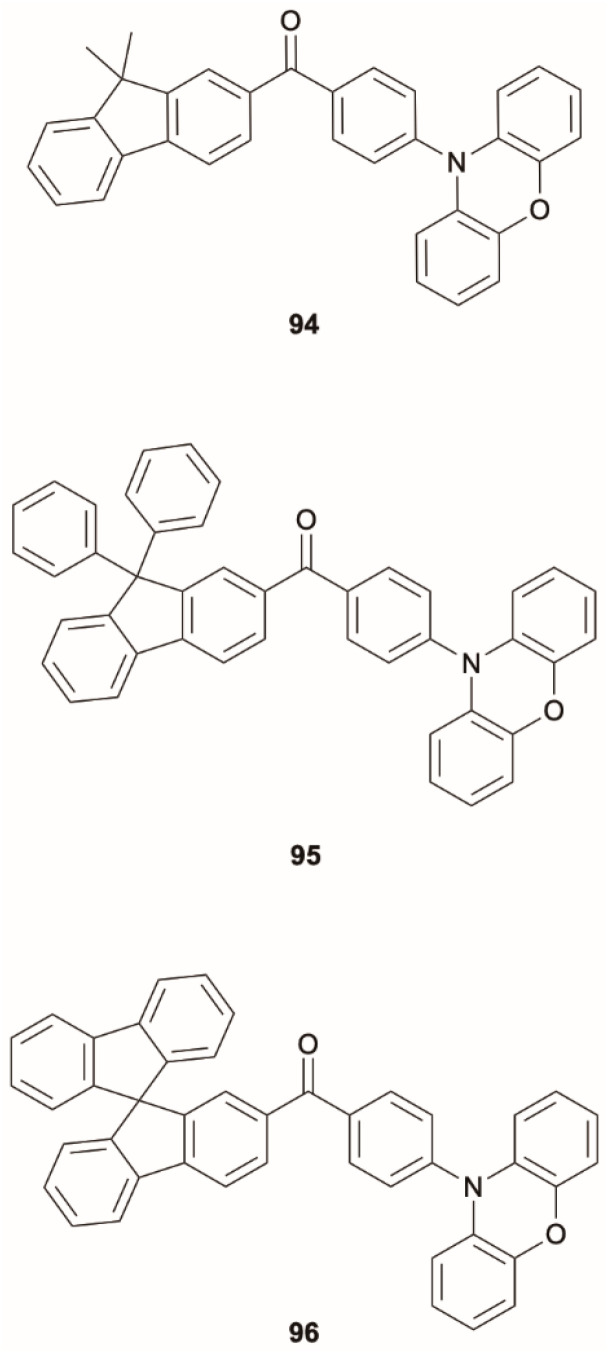
Chemical structure of compounds **94**–**96**.

Guo et al. [82] reported three donor-acceptor (D–A) compounds, **94**–**96** (Scheme 31), consisting of electron-donating phenoxazine and fluorene derivatives coupled with an electron-withdrawing benzoyl core, that are weakly emissive in solution but display DF upon aggregation. DFT/TDDFT calculations using the PBE0 functional were employed to rationalize the emission mechanism of **94** as the representative of the family. The obtained oscillator strength for S_1_ was almost zero (*f* = 0.0006), indicating that S_1_ is nonemissive. On the other hand, the optimization of the S_2_ geometry provided a relatively large *f* value (0.0523), suggesting that the efficient emission of **94** corresponds to an anti-Kasha radiative deactivation from S_2_.

**Figure 23:**
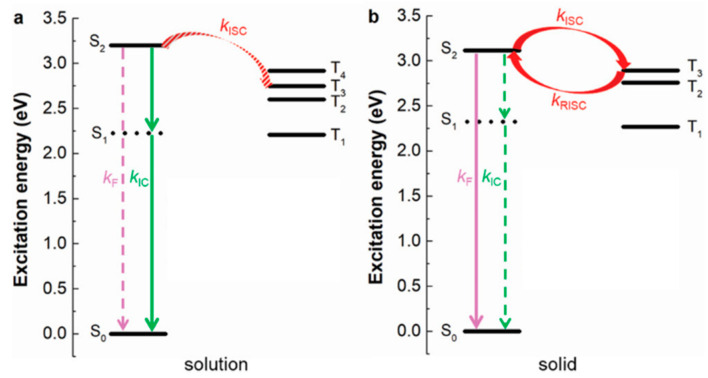
Adiabatic excitation energies for **94** (**a**) in THF solution and (**b**) in solid state. Adapted with permission from [82].

Additionally, natural transition orbital (NTO) analysis, both in THF and in solid state, has been performed. For S_2_, a typical CT state was predicted, while a significant LE feature was found for T_3_. Moreover, the small (0.22 eV) S_2_-T_3_ energy gap calculated in solid state makes an rISC process highly favorable; thus, in the solid state, T_3_ can be converted to S_2_, supporting the subsequent anti-Kasha delayed emission (see Figure 23). The full emissive mechanism in the solid state can be therefore explained by a reduced IC and a promoted ISC, with respect to the solution, resulting in the DF.

Such aggregation-induced delayed fluorescence (AIDF) appears particularly useful for OLED applications since the use of fluorescent emission, with respect to phosphorescence, allows the problem of efficiency roll-off to be reduced, while the AIE property of the compounds allows the fabrication of efficient nondoped devices. Therefore, the EL behavior of the compounds was investigated in nondoped and doped OLEDs, and external quantum efficiency, η_ext_, of up to 14.3% was obtained in the device of **95**. At 1000 cd m^−2^ the device kept excellent η_ext_ of 14.1%, and the external quantum efficiency roll-off was as small as 1.4%, indicative of the superior efficiency stability of the device based on these AIDF materials.

**Scheme 32:**
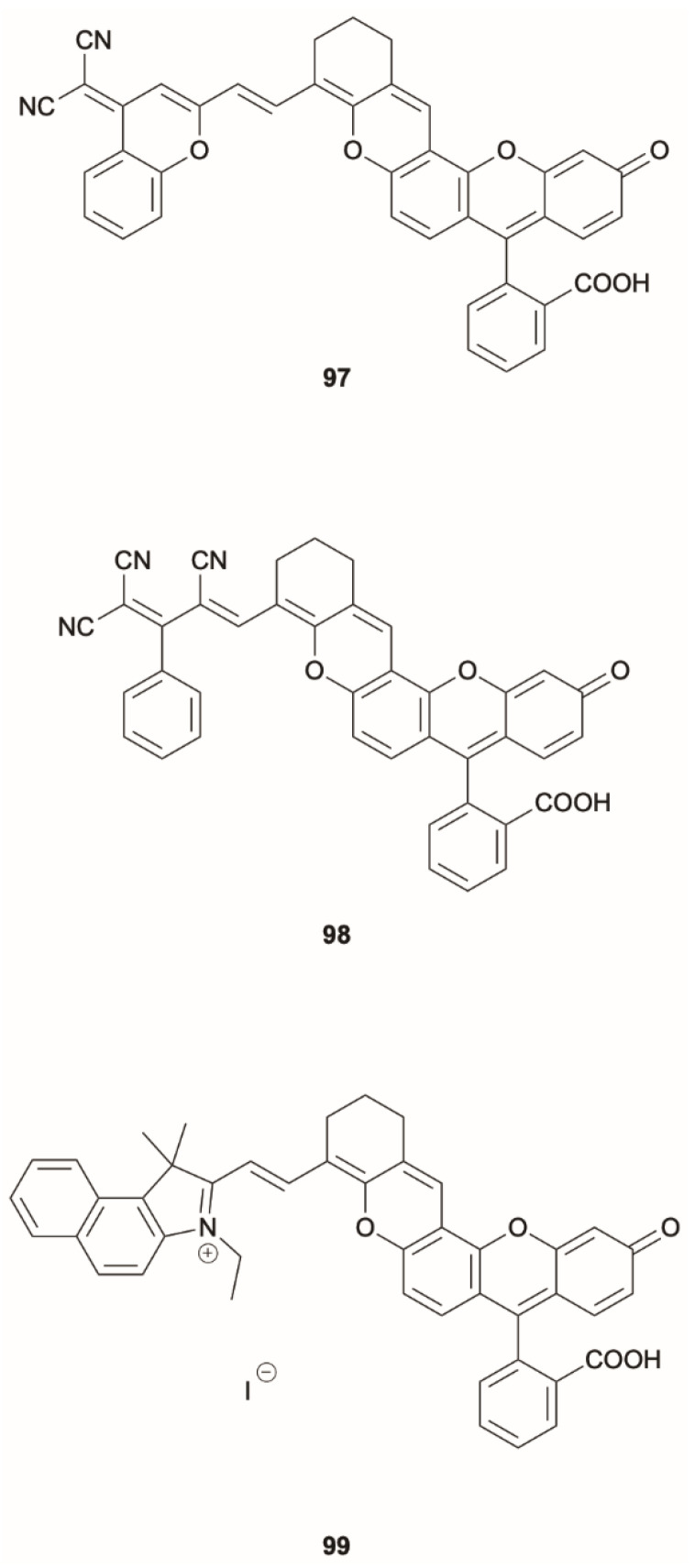
Chemical structure of compounds **97**–**99**.

**Figure 24:**
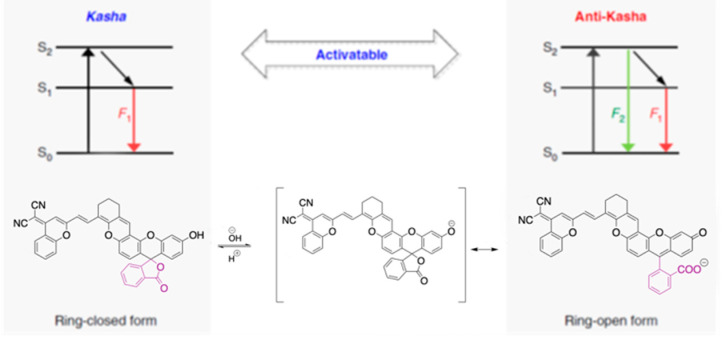
Schematic illustration of spirolactone switch-controlled **97** for tuning anti-Kasha/Kasha properties. Reprinted from [83].

Shi et al. [83] proposed a strategy to obtain ratiometric sensing for quantitative analysis of biomolecules in a dynamic cellular environment by using dyes able to switch from a close Kasha form to an open anti-Kasha one characterized by dual emission. In particular, **97** (Scheme 32), where fluorescein is covalently linked to a chromene unit, displays in solution two fluorescences at 520 and 700 nm, assigned, through DFT-TDDFT studies at M06-2X/Def2-SVP level, to emissions from S_2_ and S_1_, respectively. According to the quantum chemical calculations, a ΔE(S_2_-S_1_) equal to 0.74 eV was determined. Such a high energy gap, together with the different S_2_ and S_1_ orbital localization, the former mainly on the fluorescein fragment and the latter on the chromene moiety, justify the slow S_2_-S_1_ IC resulting in the observed dual fluorescence. For the corresponding closed form of **97**, only Kasha emission from S_1_ was recorded (see Figure 24).

**Figure 25:**
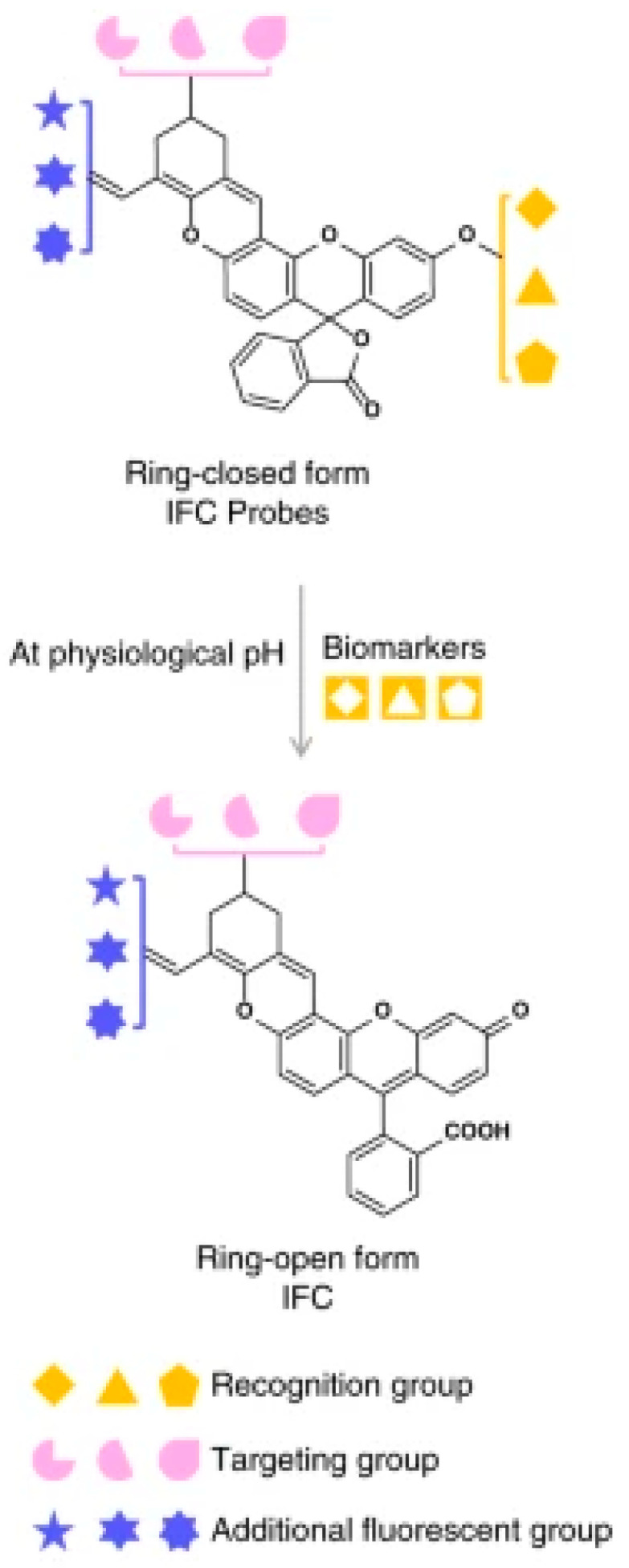
Chemical structures of **97**–**99** with different biomolecular-recognition groups as quantitative sensing platform (IFC stands for integrated fluorescein with chromene). Reprinted/adapted from [83].

Starting from **97**, similar compounds (**98**, **99**) (Scheme 32) were prepared and studied. Through D–π–A structural modifications, the ΔE(S_2_-S_1_) gap can be modulated to provide diverse anti-Kasha/Kasha chromophores exhibiting an invariant S_1_-S_0_ NIR Kasha emission and an anti-Kasha visible S_2_-S_0_ one. In the physiological pH range, these chromophores remain in their open form, emitting from both S_2_ and S_1_. A quantitative sensing platform can be realized by replacing the hydroxy group with different biorecognition units (Figure 25) When the probes in their closed form (with only the NIR emission from S_1_) encounter the corresponding analyte, the hydroxyl group is restored, turning the probes to their open form at the desirable physiological pH, with both S_2_ and S_1_ emissions. The invariant NIR Kasha emission from the S_1_ state acts as internal reference while the green emission from the anti-Kasha-active S_2_ state linearly increases with the concentration of targeted analyte. Using this strategy, the authors demonstrated the ratiometric quantification of cysteine and glutathione in living cells and animals. 

**Scheme 33:**
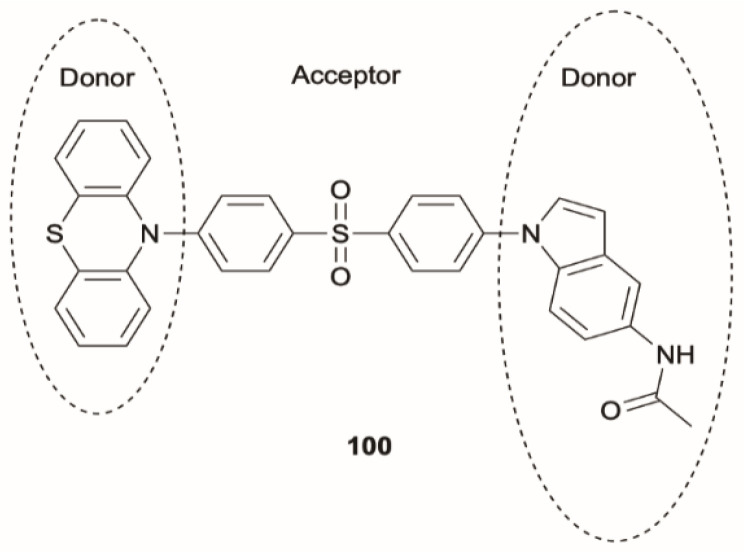
Chemical structure of compound **100**.

**Figure 26:**
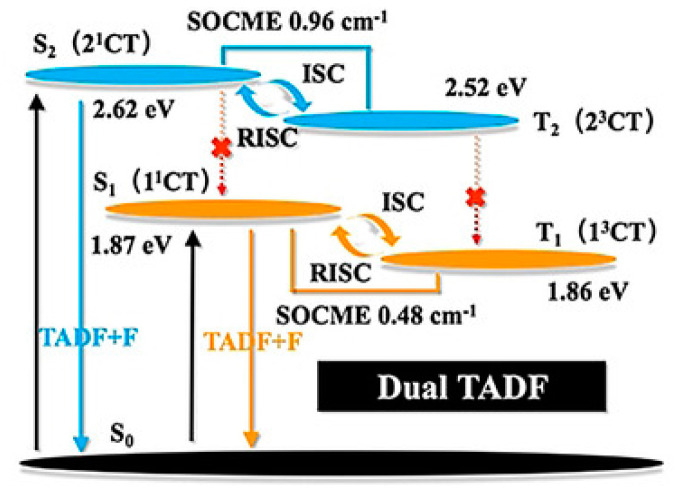
Possible mechanism for the dual TADF proposed for **100** on the basis of TDDFT calculations (SOCME represents spin-orbit coupling matrix element). Adapted with permission from [84].

Luo et al. [84] reported dual TADF emission in a single luminophore arising from anti-Kasha/Kasha pathways from different excited electronic states (see Figure 26). The authors designed the unsymmetrical D–A–D′ molecular structure **100** (Scheme 33) (where D = phenothiazine, D′ = N-(1H-indole-5-yl) acetamide and A = diphenylsulfone) which possesses two CT states localized on the D–A and D′–A subunits of the molecule, as elucidated by means of B3LYP/6-31G(d) DFT and TDDFT calculations. For each CT singlet-triplet pair, the calculated energy splitting was found to be less than 0.1 eV, allowing, both in diluted solution and in the aggregated state (THF/water, solvent/nonsolvent mixture), very fast ISC and rISC rates that produce two independent DFs with different colors and lifetimes. Due to its favorable features, **100** was successfully employed in time-resolved fluorescence cell imaging. The main advantage of this probe was its capability to provide complementary dual-channel lifetime mapping that reduces time-resolved imaging distortions. 

**Figure 27:**
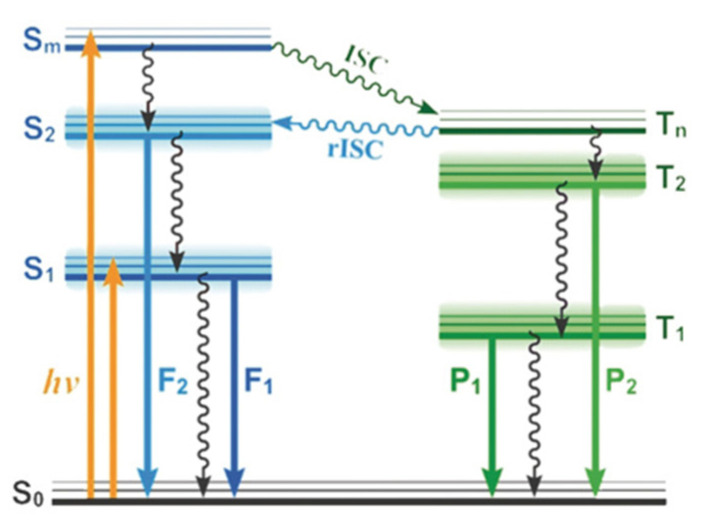
Suggested mechanism for the upper excited state fluorescence from neat solid **101**, **102** and **103**, based on DFT/TDDFT calculations. Adapted with permission from [85].

**Scheme 34:**
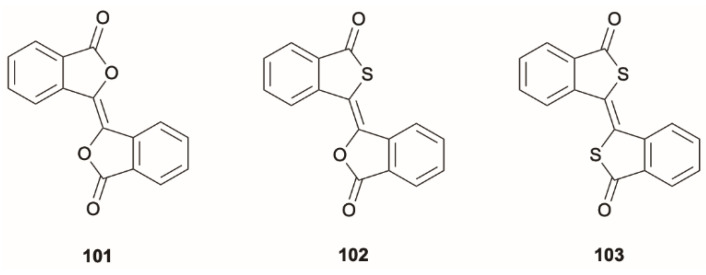
Chemical structure of compounds **101**–**103**.

Wu et al. [85] presented three heterocyclic 1,2-diphenyl ethylene derivatives, **101**–**103** (Scheme 34), that, at ambient conditions in the solid state, display excitation wavelength-dependent multiple emission, comprising prompt, delayed and RTP components originating from first and higher-energy excited singlet and triplet levels. The first and upper excited singlet and triplet states were responsible for the observed dual fluorescence and dual phosphorescence, as supported by QM/MM calculations with TDDFT excitation energies for the QM region computed at B3LYP/6-31G(d) level. In particular, prompt fluorescence from S_1_ and prompt and TADF emission from S_2_ were observed for all compounds, while dual T_2_/T_1_ RTP was recorded only for **101**, **102** and **103** displaying single RTP from T_2_ (see Figure 27).

For **101**, an S_2_ level of mainly (π, π*) character, energetically close to triplet states of mixed (*n*, π*)/(π, π*) nature, and an S_1_ and its close triplets of (π, π*) symmetry were calculated. For the other two compounds, an (*n*, π*) S_2_ level close to (π, π*) triplets together with an S_1_ and its adjacent triplets of (π, π*) symmetry were computed. Therefore, based on symmetry considerations and the small E(S_2_-T_n_) (0.08, 0.04 and 0.02 eV for **101**, **102** and **103**, respectively), an easy DF from S_2_ and a negligible one from S_1_ were predicted. Moreover, the large ΔE(T_2_-T_1_) of **101**, 1.40 eV, explains its observed dual RTP.

**Scheme 35:**
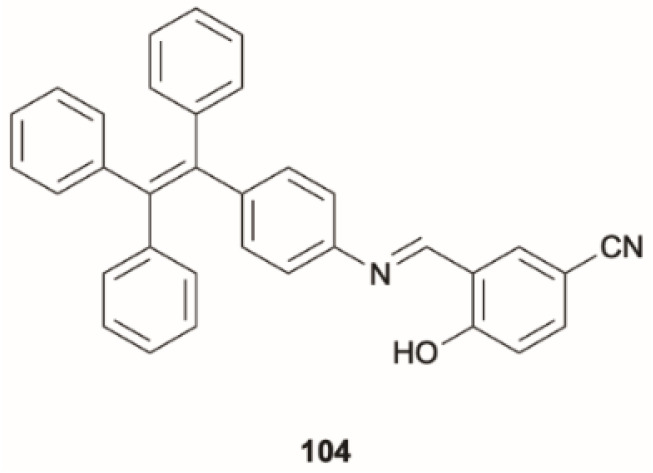
Chemical structure of compound **104**.

**Figure 28:**
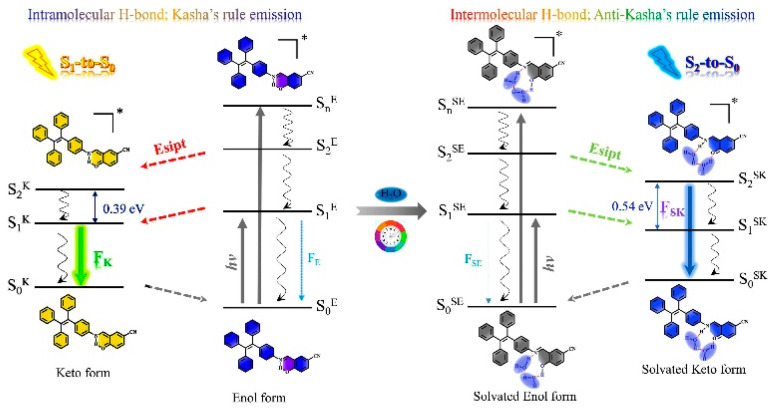
Possible mechanism for the dynamic stimuli-responsive fluorescence switch. S_0_^E^: ground state of enol form of **104**; S_n_^E^: excited state of enol form of **104**; S_0_^K^: ground state of keto form of **104**; S_n_^K^: excited state of keto form of **104**; S_0_^SE^: ground state of enol form of solvated **104**; S_n_^SE^: excited state of enol form of solvated **104**; S_0_^SK^: ground state of keto form of solvated **104**; S_n_^SK^: excited state of keto form of solvated **104**. Adapted from [86], Copyright 2021, with permission from Elsevier.

Sun et al. [86] reported a tetraphenylethene-substituted Schiff base, **104** (Scheme 35), which exhibits water-induced fluorescent switch from weak yellow-green Kasha emission to intense sky blue anti-Kasha one. According to TDDFT calculations at the B3LYP/6-31G(d) level of theory, in nonprotic solvents, an ESIPT process involving an intramolecular O-H^…^N hydrogen bond is expected to lead to a ketone structure, responsible for the Kasha yellow emission (see Figure 28). When water was added, an intramolecular hydrogen bond was broken in favor of an intermolecular one with a water molecule. ESIPT associated with this interaction leads to a solvated ketone structure whose 440 nm emission was assigned to radiative deactivation from S_2_, and the S_2_-S_1_ IC was inhibited by the large ΔE(S_2_-S_1_), 0.54 eV. It should be underlined that this ESIPT process is correctly included in the present review, despite what was mentioned in the introduction, since it results in the formation of a new species from which anti-Kasha emission is observed. A practical use of **104** in rewritable papers and fluorescent sensors was developed with potential applications in information security protection, multilevel anticounterfeiting and data storage.

**Scheme 36:**
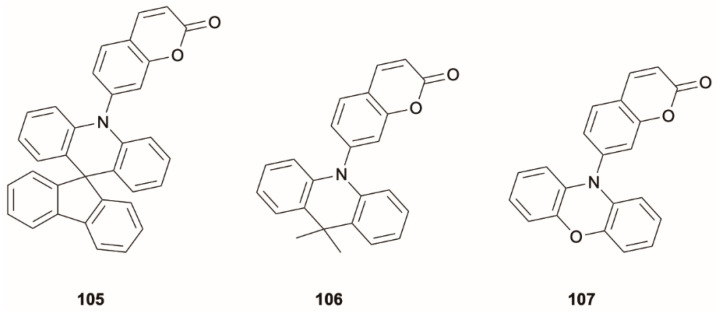
Chemical structure of compounds **105**–**107**.

**Figure 29:**
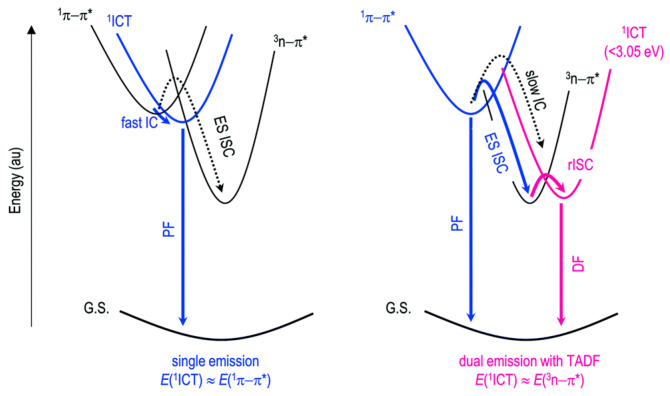
Mechanism proposed for the single Kasha D-coumarin derivatives (**left**) and dual anti-Kasha emission (**right**) from **105**, **106** and **107**. Abbreviations: G.S., ground state; ES ISC, El-Sayed-rule-allowed ISC; PF, prompt fluorescence; DF, delayed fluorescence. Adapted from [87], Copyright 2021, with permission from the Royal Society of Chemistry.

Due to the presence of (π, π*) and (*n*, π*) levels, coumarin exhibits efficient El-Sayed-allowed ISC and therefore represents a useful scaffold to populate triplet levels in the absence of heavy atoms [87]. Jhun et al. [87] investigated a series of D-coumarin derivatives where D represents units with different oxidation potentials. Among the studied compounds, 10*H*-spiro(acridine-9,9′-fluorene), **105**; 9,9′-dimethyl-9,10-dihydroacridine, **106**; and 10*H*-phenoxazine, **107** (Scheme 36), display anti-Kasha dual emission in toluene solution comprising a high-energy fluorescence at 420 nm of (π, π*) with partial CT character and a low-energy one in the 470–580 nm region of ICT character. Steady-state and time-resolved results revealed the TADF nature of the low-energy emission, which involves thermal repopulation of the ^1^ICT state from a close ^3^(*n*, π*) one. This latter is populated through El-Sayed-allowed ISC from the ^1^(π, π*) level since both states are localized on the coumarin moiety (see Figure 29). Intriguingly, such dual fluorescence is produced from (π, π*) and ICT transitions, both usually characterized by high oscillator strength. On the contrary, a single Kasha fluorescence was observed for the reference carbazole-coumarin compound, where the low (π, π*)-ICT energy gap (0.23 eV), smaller than those of the other investigated D-coumarin derivatives, allows a faster IC to the ^1^ICT state rather than ISC to the ^3^(*n*, π*) one. It has to be noted that TDDFT calculations on this series of compounds, aimed at investigating the nature of the emissive state(s), revealed that the B3LYP functional provided results closer to the experimental values with respect to other tested (CAM-B3LYP and PBE0) functionals.

The dual fluorescence was successfully used by the authors for ratiometric temperature monitoring in the 120–300 K range and triplet quencher detection. 

**Scheme 37:**
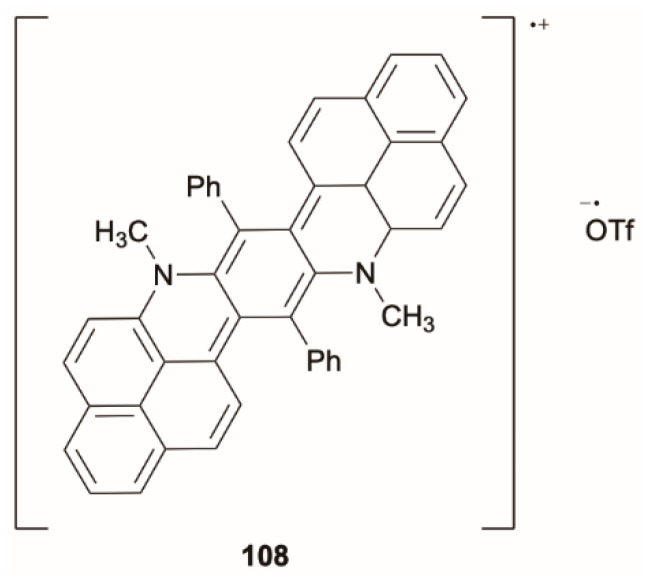
Chemical structure of compound **108**.

Imran et al. [88] reported on the anti-Kasha behavior of radical cation **108** (Scheme 37). When excited at 537 nm, DCM solutions of **108** display a red fluorescent emission centered at about 634 nm (Φ = 9.3%), which is intensified (Φ = 19.3%) when the compound is dispersed in PMMA matrix (1 wt%). The compound possesses one of the highest photostabilities among radical emitters with a half-life of 9.5 × 10^4^ s under 350 nm irradiation. This property was ascribed to its cationic charge, which stabilizes ground and excited state molecular orbitals and inhibits their oxidation. DFT and configuration interaction singles calculations at the UB3LYP/6-31G+(d,p) level provided a low-energy excited doublet. According to the energy gap law, nonradiative deactivation was predicted from this state due to its closeness to the ground state. Therefore, the observed emission was associated with higher-energy calculated doublets, in violation of the Kasha rule.

**Scheme 38:**
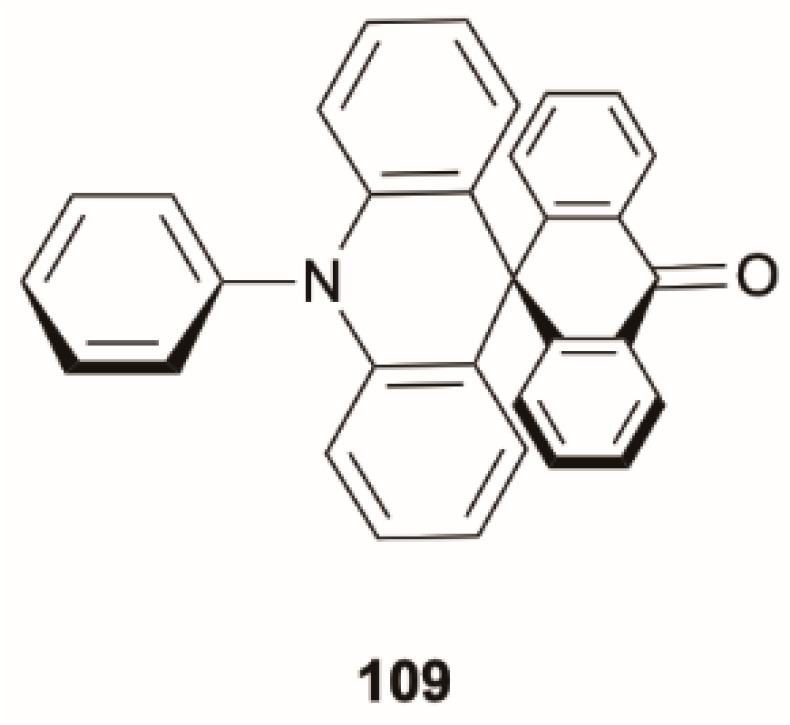
Chemical structure of compound **109**.

**Figure 30:**
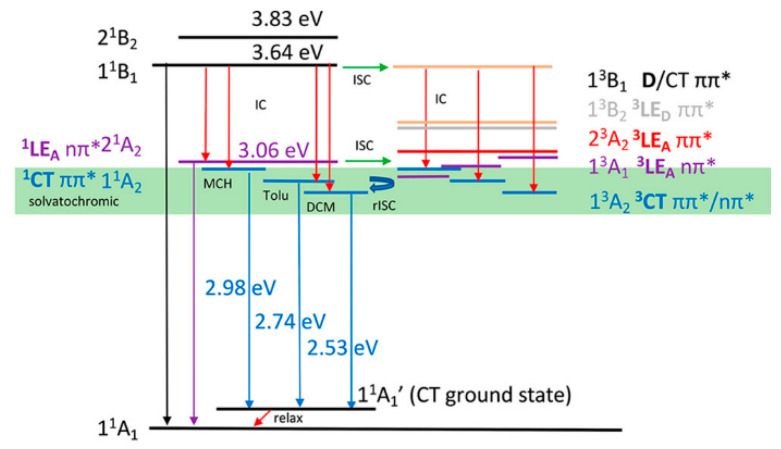
Measured energy levels from spectral onsets of **109** in solvents of different polarity. The green band represents the energy range over which solvatochromic states were observed. Red arrows represent nonradiative transitions. Adapted with permission from [89], Copyright 2021, American Chemical Society, https://pubs.acs.org/doi/10.1021/acs.jpclett.0c03314 (accessed on 8 July 2021). Further permissions related to the material excerpted should be directed to the American Chemical Society.

Multiple anti-Kasha emissions from three singlet states of a single molecule were reported by Franca et al. [89] for a spiro compound, 10-phenyl-10*H*,10′*H*-spiro[acridine-9,9′-anthracen]-10′-one, **109** (Scheme 38), characterized by rigidly connected orthogonal D–A units. Excitation of toluene solutions of **109** at high energy provides simultaneous emission from three states (see Figure 30) which were described, based on previously reported calculations on this molecule [90], performed at the DFT/MRCI-R/SV(p) level, as LE_D_ (π, π*) (^1^B_1_ in Figure 30), LE_A_ (*n*, π*) and CT (π, π*) states (in order of decreasing energy), the first two being locally excited states pertaining to the acridine (D) and the anthracenone (A) moieties, respectively. The lower-energy CT state, very close in energy to LE_A_, provides both prompt and delayed fluorescence. The strongest allowed transition was ascribed to the high energy state, LE_D_, located at about 0.6 eV above the other two. Emission from LE_D_ competes with both IC (to LE_A_ and CT lower-lying states) and ISC to a close triplet state which then decays to populate a lower triplet state yielding DF from rISC. Direct population of the weaker states produces both prompt (from LE_A_ and CT states) and delayed (from ISC between LE_A_ and ^3^LE_A_ followed by rISC to CT state) emissions. 

The existence of the three emissions was unequivocally assessed by working in aerated/deaerated conditions and by varying both the excitation wavelength, resulting in a change in the relative intensities of the emissions, and the solvent. Strong solvatochromic relaxation was observed for the lowest-energy singlet CT state, while the close-lying LE_A_ state was unaffected by polarity. The origin of such unprecedented rich photophysics of **109** was attributed to its rigid perpendicular geometry which effectively electronically decouples the D and A moieties. This ensures small ΔE_ST_ values and activates the TADF from a molecular CT state, in addition to the prompt emissions from the local LE_D_ and LE_A_ states.

## 9. Conclusions

This review of the literature of the past 10 years collects examples of purely organic compounds exhibiting photophysical behaviors that violate the Kasha rule. Mechanisms involved in different systems are frequently related to the same molecular properties, which are a large E_n_-E_n−1_ energy gap, a strong E_n_-E_0_ oscillator strength and a weak E_n−1_-E_0_ oscillator strength (*n* > 1), where E refers to both singlet and triplet levels. Moreover, symmetry considerations on E_n_ and E_n−1_ have to be taken into account, together with the energy gap ones, as a factor that inhibits E_n_-E_n−1_ IC, making the radiative deactivation from E_n_ competitive. From the collected examples it appears that even a ΔE(E_n_-E_n−1_) as small as 0.35 eV is enough to favor emission from E_n_. This value is derived from computational studies, some of which can be of course further improved. However, at this stage, a ΔE(E_n_-E_n−1_) smaller than the generally accepted 1 eV limit could be enough to observe anti-Kasha emission.

Alternatively, thermally activated anti-Kasha behavior may occur in the presence of a small E_n_-E_n−1_ energy gap (about 0.2 eV) by both reverse IC and reverse ISC. 

In addition to basic research considerations, anti-Kasha behavior might offer improved performances useful for many practical applications. Among them, dual fluorescence from S_2_ and S_1_ and/or phosphorescence from T_2_ and T_1_ allow the development of OLEDs with dual electroluminescence based on a single compound, strongly simplifying device technology and concomitantly reducing fabrication costs. Other interesting issues are the intrinsic response of dual emitters to external stimuli such as temperature, allowing their use as fluorescence thermometers, or water, being optimal as humidity sensors, or encryption/decryption. The different lifetimes of their emitting states offer unprecedented opportunities for their use in time-resolved bio-imaging, solving the problem of detection in weak emission areas.

## Abbreviations

IC = internal conversion; ISC = intersystem crossing; RISC or rISC = reverse intersystem crossing; FRET = fluorescence resonance energy transfer; DFT = density functional theory; TDDFT = time-dependent density functional theory; CASPT2 = complete active space second-order perturbation theory; MRCI = multireference configuration interaction; ICT = intermolecular charge transfer; CT = charge transfer; AIE= aggregation-induced emission; CIE = crystallization-induced emission; QY = quantum yield; PMMA = polymethylmetacrilate; RT = room temperature; RTP = room temperature phosphorescence; DCM = dichloromethane; TA = transient absorption; EL = electroluminescence; DF = delayed fluorescence; TADF = thermally activated delayed fluorescence; TAPC = 1,1′-bis(di-4-tolylaminophenyl)cyclohexane; TmPyPB = 1,3,5-tri(m-pyrid-3-yl-phenyl)benzene.
